# Detection and diversity of the mannosylerythritol lipid (MEL) gene cluster and lipase A and B genes of *Moesziomyces antarcticus* isolated from terrestrial sites chronically contaminated with crude oil in Trinidad

**DOI:** 10.1186/s12866-021-02419-4

**Published:** 2022-02-04

**Authors:** Amanda C. Ramdass, Sephra N. Rampersad

**Affiliations:** Biochemistry Research Laboratory (Rm216), Department of Life Sciences, Faculty of Science and Technology, The University of the West Indies, St. Augustine, West Indies Trinidad and Tobago

**Keywords:** Mannosylerythritol lipids, Biosurfactant, Glycolipid, Lipase, *Moesziomyces antarcticus*, Phylogeny

## Abstract

**Background:**

Mannosylerythritol lipids (MELs) belong to the class of glycolipid biosurfactants and are produced by members of the Ustilago and Moesziomyces genera. Production of MELs is regulated by a biosynthetic gene cluster (MEL BGC). Extracellular lipase activity is also associated with MEL production. Most microbial glycolipid-producers are isolated from oil-contaminated environments. MEL-producing yeast that are capable of metabolizing crude oil are understudied, and there is very limited data on indigenous strains from tropical climates. Analysis of the MEL BGC and lipase genes in Trinidad *M. antarcticus* strains, using a gene-targeted approach, revealed a correlation between their intrinsic capability to degrade crude oil and their adaptation to survive in a chronically polluted terrestrial environment.

**Results:**

*M. antarcticus* was isolated from naturally-occurring crude oil seeps and an asphaltic mud volcano in Trinidad; these are habitats that have not been previously reported for this species. Genus identification was confirmed by the large-subunit (LSU) and the small-subunit (SSU) sequence comparisons and species identification was confirmed by ITS sequence comparisons and phylogenetic inference. The essential genes (*Emt1, Mac1, Mac2, Mmf1*) of the MEL BGC were detected with gene-specific primers. Emt1p, Mac1p and Mmf1p sequence analyses confirmed that the Trinidad strains harboured novel synonymous amino acid (aa) substitutions and structural comparisons revealed different regions of disorder, specifically for the Emt1p sequence. Functionality of each protein sequence was confirmed through motif mining and mutation prediction. Phylogenetic relatedness was inferred for Emt1p, Mac1p and Mmf1p sequences. The Trinidad strains clustered with other *M. antarcticus* sequences, however, the representative Trinidad *M. antarcticus* sequences consistently formed a separate, highly supported branch for each protein. Similar phylogenetic placement was indicated for *LipA* and *LipB* nucleotide and protein sequences. The Trinidad strains also demonstrated lipolytic activity in culture, with an ability to utilize different carbon sources. Comparative evolution of MEL BGC and *LipA* gene suggested early and late duplication events, depending on the gene, followed by a number of speciation events within *Ustilaginaceae*. *M. antarcticus* and *M. aphidis* were separated from all other members of *Ustilaginaceae* and two gene homologues were detected, one for each species.

**Conclusions:**

Sequence analyses was based on a novel gene-targeted approach to analyze the essential genes of the MEL BGC and *LipA* and *LipB* genes of *M. antarcticus* strains from Trinidad. The findings indicated that these strains accumulated nucleotide mutations to a threshold level that did not affect the function of specific proteins encoded by the MEL BGC and *LipA* and *LipB* genes. The biosurfactant and lipase enzymes secreted by these Trinidad *M. antarcticus* strains facilitated their survival in oil-contaminated terrestrial environments. These findings suggest that the Trinidad strains should be explored as promising candidates for the commercial production of MEL biosurfactants and lipase enzymes.

**Supplementary Information:**

The online version contains supplementary material available at 10.1186/s12866-021-02419-4.

## Background

Different microbes are capable of producing extracellular amphipathic compounds that serve as biosurfactants. These surface-active compounds are structurally diverse, and their core functions are to reduce interfacial tension, increase the solubility and surface area contact to increase the rate of phase transfer and bioavailability of insoluble compounds [1, 2]. Biosurfactants increase the availability of hydrophobic nutrients to microbes thereby improving their competitiveness and survival outcome in saturated non-polar environments. This is especially important to degradation of petroleum hydrocarbons which are degraded slowly because they are extremely recalcitrant hydrophobic compounds [1]. There are physical, chemical and biological approaches to remediation of oil-contaminated soil, however, only microbial bioremediation utilizes renewable organic resources and low cost technology [3, 4]. Microbial biosurfactants offer select advantages compared to their chemical counterparts, e.g. low toxicity, environmentally compatible, biodegradable, higher stability in different environmental conditions, and are non-hazardous [5–8]. The combined action of microbial biodegradation with biosurfactant activity can be twice as efficient as either effect alone [9]. Biosurfactants produced by yeast and fungi are less known compared to those of bacterial origin. Biosurfactants, as tensio-active molecules, are useful in a range of industrial applications including bioremediation, agriculture, detergent, medical, pharmaceutical, food, textile, paint, leather, paper, mining, nanotechnology, and bioprocessing industries [3].

*Moesziomyces antarctica* was originally isolated as an obligate psychrophile inhabiting the 9 m-deep sediment of Lake Vanda in Antarctica [10]. It has also been found in soil, on plant surfaces where it provides secondary protection against powdery mildew infection [11, 12]. Some strains have been reported in opportunistic human infections in immunocompromised individuals [13]. The genus *Pseudozyma* is polyphyletic and member species cluster with teleomorphic species of *Ustilago, Sporisorium* and *Moesziomyces* in the *Ustilaginaceae* family [14]. As a result of the many taxonomic revisions for the majority of the ustilaginomycetous yeasts according to the ‘One Fungus = One Name’ standard [15–17] the genus name ‘*Pseudozyma*’ is no longer used [14, 18].

**Fig. 1:**
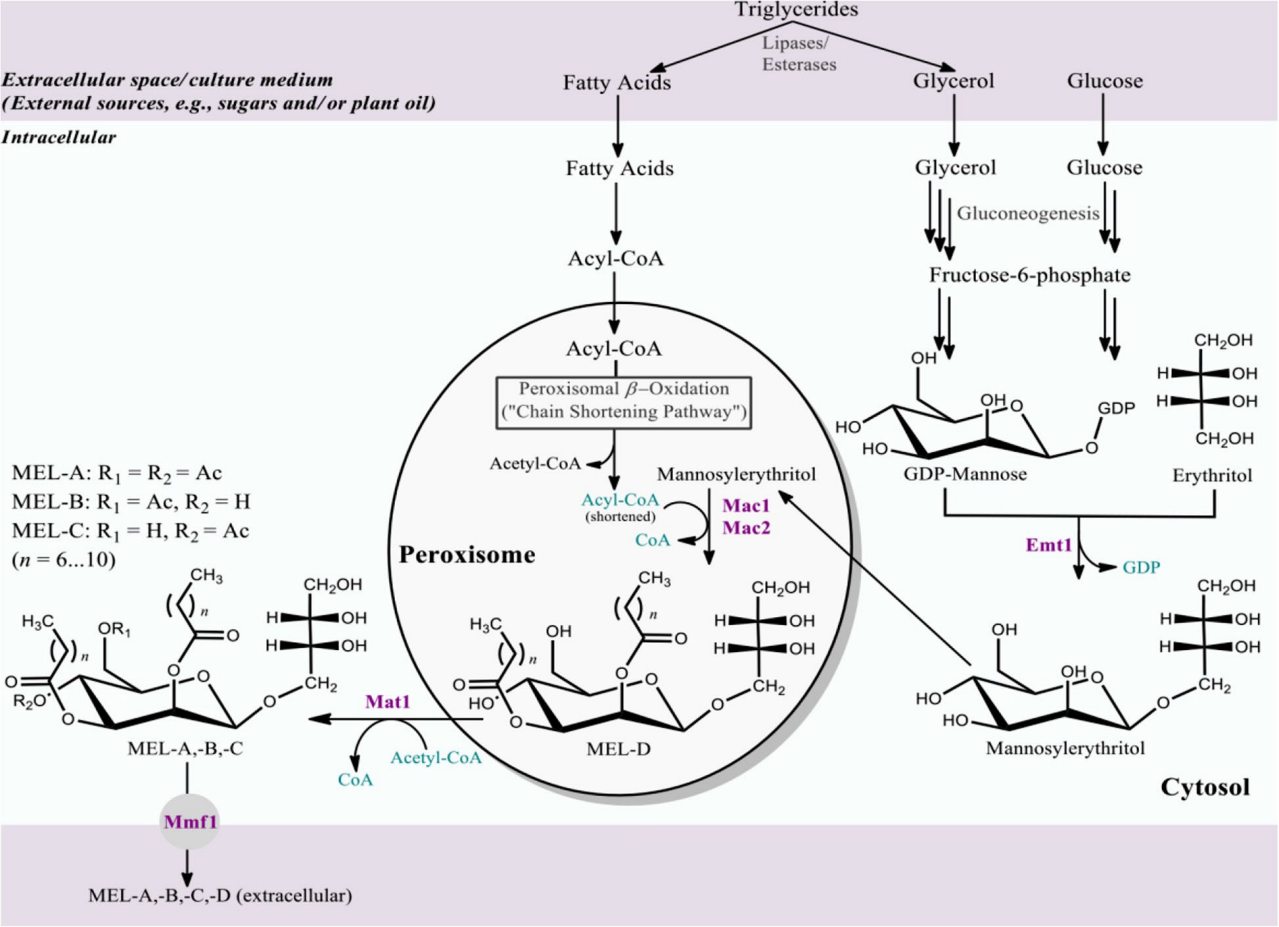
Mannosylerythritol lipids biosynthesis highlighting key metabolic pathways. The five genes include an erythritol/mannose transferase *Emt1*, two acyltransferases *Mac1* and *Mac2*, an acetyltransferase *Mat1* and a putative transporter *Mmf1*. Pathways and enzymes were derived from the KEGG database for *U*. *maydis* and adapted and combined with the MEL synthesis pathway from Hewald, et al. [29] and Saika, et al. [27]

Mannosylerythritol lipids (MELs) belong to the class of glycolipid biosurfactants [19] and their structure consists of 4-*O*-*β*-D-mannopyranosyl-*meso*-erythritol as the hydrophilic moiety, and fatty acids as the hydrophobic moiety [20]. MELs can be differentiated according to the acetylation pattern on the core structure [21] (Fig. 1). MELs are secreted by a number of different fungal species, e.g. *Ustilago maydis*, *M. antarctica* T-34 and JCM10317, *M. aphidis* DSM70725 and *M. hubeiensis* SY62, *Schizonella melanogramma*, *Kurtzmanomyces* sp. [20, 22–26]. Strains of *M. antarctica*, *M. parantarctica*, *M. aphidis* and *M. rugulosa* produce primarily MEL-A; *M. graminicola*, *M. hubeiensis*, *M. siamensis* and *M. shanxiensis* produce MEL-C and *M. tsukubaensis* produce a diastereomer type of MEL-B [21, 27]. MEL-producers can be phylogenetically placed according to characteristics of MEL formation and rRNA sequence identity [28].

The industrial applications of MELs were assessed over the last 50 years since their discovery in the 1950’s [30–34]. MELs have demonstrated antimicrobial activity, and are involved in attachment inhibition and biofilm dispersal which prevent microbial adhesion and desorption in biofilm formation [35]. Additionally, MELs have demonstrated biological activities such as neuronal differentiation in mammalian PC12 cells [36], inhibition of dopamine receptors [37], high affinity to immunoglobulins [38], and they increase the transfection efficiency of liposomes [39]. MELs have gained the reputation as environmentally-friendly biosurfactants with superior surface-active properties [21, 33, 40, 41].

In *U*. *maydis* and *M. antarcticus*, the MEL biosynthetic pathway is catalysed by five enzymes based on predicted function of the primary protein sequence: an erythritol-mannosyl transferase (Emt1p) which drives the first committed step in MEL synthesis and is essential for MEL production; two mannose/acyltransferases (Mac1p and Mac2p), an acetyltransferase (Mat1p) and a mannosylerythritol lipid transporter (Mmf1p), all of which are encoded by genes arranged in a tightly-regulated biosynthetic gene cluster (BGC) [25, 29, 42, 43]. Emt1p is localized to the cytosol where precursor saccharides are abundant and where the transfer of glycosyl group is located; Macp1 and Mac2p localize these enzymes to peroxisomes where peroxisomal β-oxidation of fatty acids and transfer of short- and medium-chain fatty acids to the C-2 and C-3 positions of mannosylerythritol occur; Mat1p is an acetyl-CoA-dependent MEL acetyltransferase; all structural variants of MEL (A to D) are extracellularly secreted and detected, therefore, Mmf1p is a membrane-bound transporter localized to the plasma membrane and demonstrates substrate specificity [44, 45]. For *M. antarcticus,* the MEL BGC was located towards the terminal end of scaffold 19 which corresponded to chromosome 7 of *U. maydis* [46]. There is also a rearrangement of the *PaEmt1* and *PaMac1* genes of *M. antarcticus* when compared with *U. maydis*. Differential patterns of gene expression of the MEL BGC in *M. antarcticus* and *U. maydis*, depending on the carbon source, suggested that *M. antarcticus* may be able to produce MELs under oleaginous conditions [42].

*M. antarcticus* produces and secretes high amounts of extracellular lipases (EC3.1.1.3 triacylglycerol lipase, lipase A and B) which can be purchased commercially [47]. Two genes, *PaLipA* and *PaLipB*, were isolated from *M. antarcticus* strain T-34 and strain 1E5 were upregulated to exhibit higher lipase activity which accelerated oil metabolism [48]. Iterative saturation mutagenesis (ISM) and studies on directed evolution of *LipA* and *LipB* have provided information on the role of key residues and protein subdomains in maintaining thermostability, active site conformation, enantioselectivity, and substrate specificity of these secreted lipases [49]. PaLipAp and PaLipBp contain 462 and 342 amino acids (aa), respectively, and both protein structures contain an α/β hydrolase-fold; an active site triad located at Ser184, Asp334, and His366 in PaLipAp and Ser105, Asp187, and His224 in PaLipBp [50]. Multiple mutations in PaLipAp and PaLipBp are deleterious to enzyme activity. Understanding aa substitutions is important to bio-engineering the translated protein [49]. Importantly, Saika, et al. [51] confirmed that MEL biosynthesis is required for extracellular lipase production and secretion in *M. antarcticus* strain GB-4. A study of MEL BGC of *M. antarcticus* isolates should, therefore, include simultaneous analysis of genes that encode extracellular lipases for oil/lipid metabolism [52].

Trinidad is the largest producer of oil and natural gas in the Caribbean and has a long history of exploration and production which began in 1857 [53]. In Trinidad, the main inland hydrocarbon region is located in the southern part of the island. Long-term oil discharge into the terrestrial environment occurs in the form of natural seeps and leaking pipelines. It is hypothesized that these Trinidad isolates would (i) have the capability to utilize crude oil as a carbon source as a consequence of long-term adaptation to chronic contamination, (ii) demonstrate the ability to produce biosurfactants (MELs), (iii) possess extracellular secreted lipase activity as a result of expression of the *PaLipA* and *PaLipB* genes, and (vi) display a high level of conservation of aa sequences of all genes constituting the MEL BGC as well as PaLipAp and PaLipBp sequences. The atypical habitat of crude oil-saturated soil inhabited by the *M. antarcticus* Trinidad strains, also suggests that there may be strain-specific changes as adaptive mechanisms of survival in these harsh environments. It is hypothesized that the Trinidad strains are phylogenetically related to other *M. antarcticus* strains based on rDNA, LSU and SSU non-coding sequences. Individual genes of the MEL BGC and lipase genes may show a higher level of genetic diversification as a result of environmental adaptation and carbon resource use. The study objectives were to develop a gene-targeted approach to (i) detect the presence of the MEL BGC and *PaLipA* and *PaLipB* genes in *M. antarcticus* Trinidad strains, (ii) compare the level of conservation of the deduced translated aa sequences encoded by the MEL BGC and by the *PaLipA* and *PaLipB* genes, and (iii) to assess the phylogenetic relationships of these proteins among *M. antarcticus* and related genera. Extracellular lipase activity of the *M. antarcticus* Trinidad strains in culture was also assessed using two different carbon sources.

## Results

### Identification of isolates

**Fig. 2:**
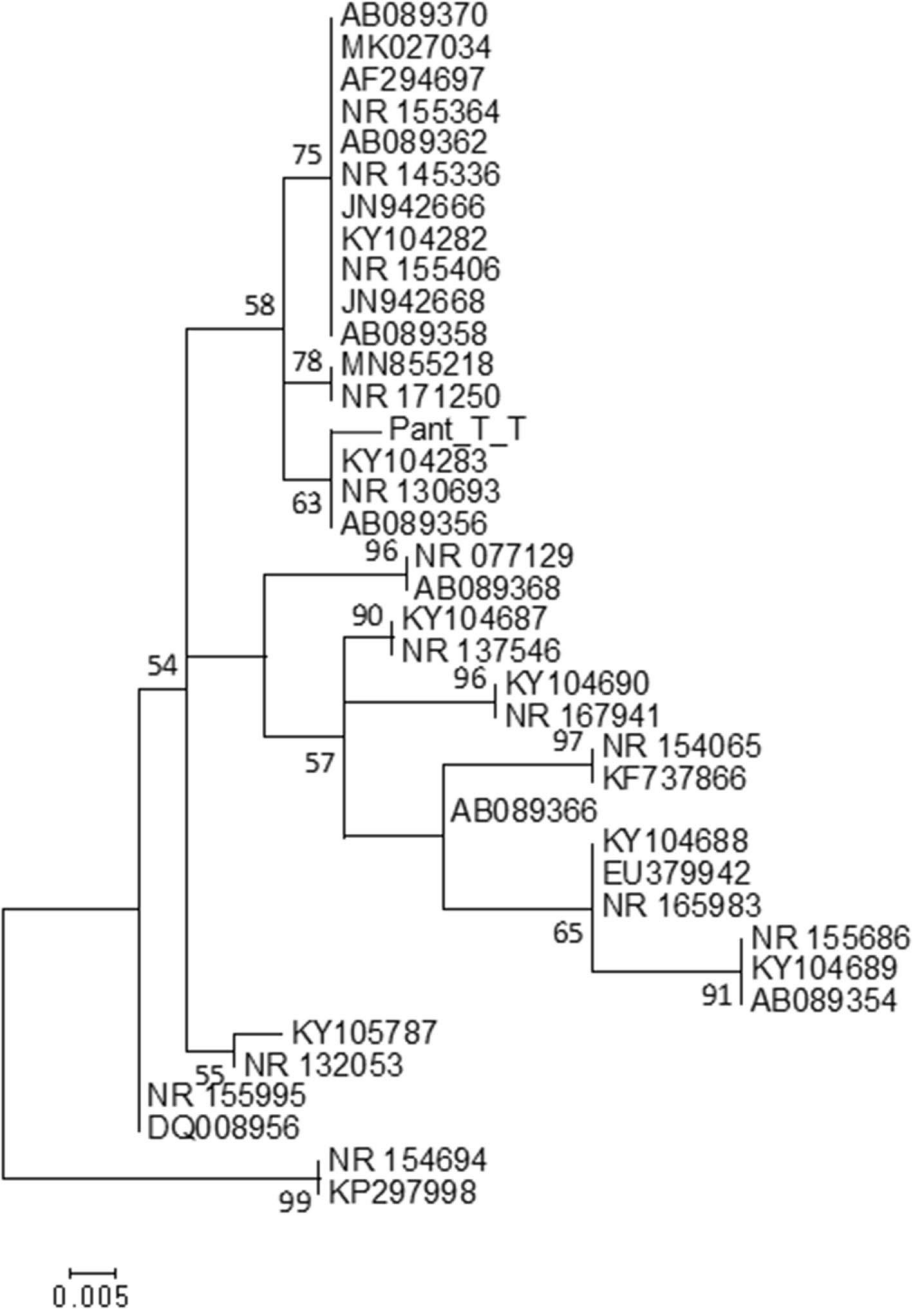
Phylogenetic analysis of aligned ITS nucleotide sequences. The representative Trinidad strain is highlighted in blue (GenBank Accession No. MZ143989)

The LSU and SSU sequence comparisons to type strains confirmed the genus of the Trinidad strains as belonging to *Moesziomyces*. ITS nucleotide sequence comparisons to type strains confirmed the identity of the Trinidad strains at the species level as *M. antarcticus*. A 50% consensus Maximum Likelihood (ML) tree of the aligned ITS sequences was hypothesized (Fig. 2). The tree was modelled for 39 sequences with 527 nucleotide sites. The evolutionary history was inferred by using the ML method based on best-fit model according to Bayesian Information Criterion (BIC) which was determined to be K2P + G4. The tree with the highest log likelihood (− 2302.587871) is shown and is drawn to scale, with branch lengths measured in the number of substitutions per site. All positions containing gaps and missing data were eliminated. Two sequences of *Anthracocystis* sp. were used as outgroups. The Trinidad representative consensus sequence was closely related to KY104283 (Strain: CBS10005; host, “*Homo sapiens*”; country: Thailand), NR_130693 (Strain and ex-type culture of “*Pseudozyma parantarctica*”: JCM 11752; host: not available; country: Thailand) and AB089356 (Strain: M9932, identical to JCM 11752; host: “*Homo sapiens*”; country: Thailand) and formed a discrete *M. antarcticus* clade with high bootstrap support (bs > 90%). *M. rugulosa* and *M. aphidis* clustered with other *M. antarcticus* sequences in a highly supported clade with evidence of polytomic branching. This indicated that ITS sequences were too invariant to enable resolution of these sequences at the species level. All other taxa were placed into species-specific clades with high bootstrap support with polytomic branching of taxa in each clade. This indicated that ITS sequences of all other taxa were separated at the inter-specific level.

### MEL genes and protein sequence analyses

The *Emt1*, *Mac1*, *Mac2* and *Mmf1* genes were successfully amplified in the Trinidad strains using the gene-specific primer pairs designed in this study. The deduced protein sequences were then analysed.

### Emt1 protein sequence analysis

**Fig. 3:**
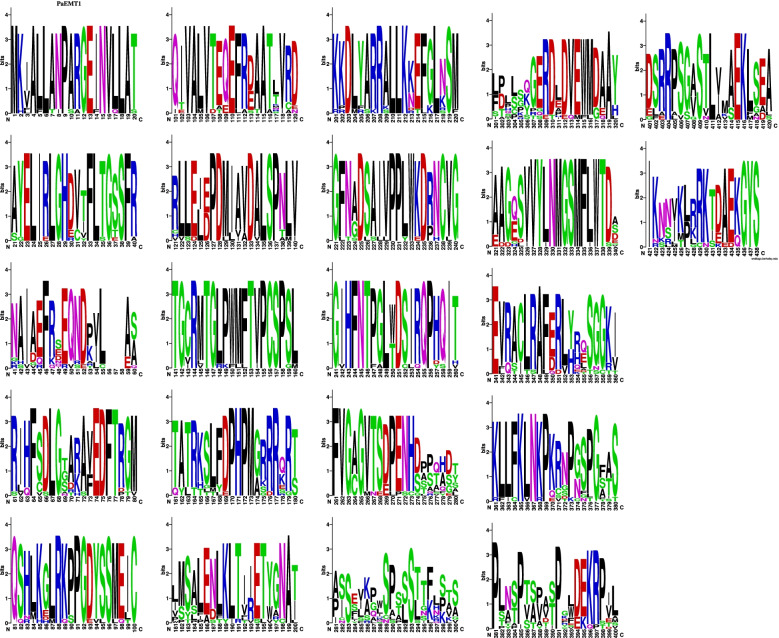
An Emt1p sequence logo is a graphical representation of an amino acid or nucleic acid multiple sequence alignment. Each logo consists of stacks of symbols, one stack for each position in the sequence. The overall height of the stack indicates the sequence conservation at that position, while the height of symbols within the stack indicates the relative frequency of each amino or nucleic acid at that position

There were four hits to sequences of the *Moesziomyces* genus with the highest query coverage and identity and the same 0.00 E-value score. The highest percentage query coverage and identity were recorded for type/reference sequence *M. antarcticus* strain T-34 (GAC75887) with 100% query coverage and 100% sequence identity match. The other *Moesziomyces* hits were obtained for XP_014653801 (*M. antarcticus*), ETS61959 (*M*. *aphidis*) and BAI77915 (*M. antarcticus*) in descending order of percentage identity. Seventeen PaEmt1p sequences were included in the aligned dataset based on Blastp results and these were used in subsequent comparisons. A region of high variability occurred at aa274 to aa307 (Fig. 3). Prior to this region, the sequences were largely conserved with a single or double substitution pattern in most cases.

A conserved domain search confirmed the identity of the aa sequence as cl10013 (https://www.ncbi.nlm.nih.gov/Structure/cdd/cddsrv.cgi?uid=415824): Glycosyltransferase superfamily 1 and related proteins with GTB topology with distinct N- and C- terminal domains each containing a typical Rossmann fold. The two domains had high structural homology despite lower level sequence homology. The large cleft, that separated the two domains located in the catalytic centre, permit a high degree of substrate flexibility.

No signal peptide sequences were present in the aligned aa sequences and there were no transmembrane (TM) domains in any of the Trinidad sequences and in any of the sequences of four main related genera: *Moesziomyces*, *Sporisorium*, *Ustilago* and *Melanopsichium*.

The glycosylation pattern of Asn-Xaa-Ser/Thr sequons with a score > 0.5 was considered. The most commonly found sequon occurred at position aa195 which contained a motif [NATK] for the majority of sequences except for the *Moesziomyces* sequences for which this glycosylation motif was absent. Another motif located at aa293 [NSTS] was predicted for only two *Ustilago* sequences: SAM82152 and CCF52717.

**Fig. 4:**
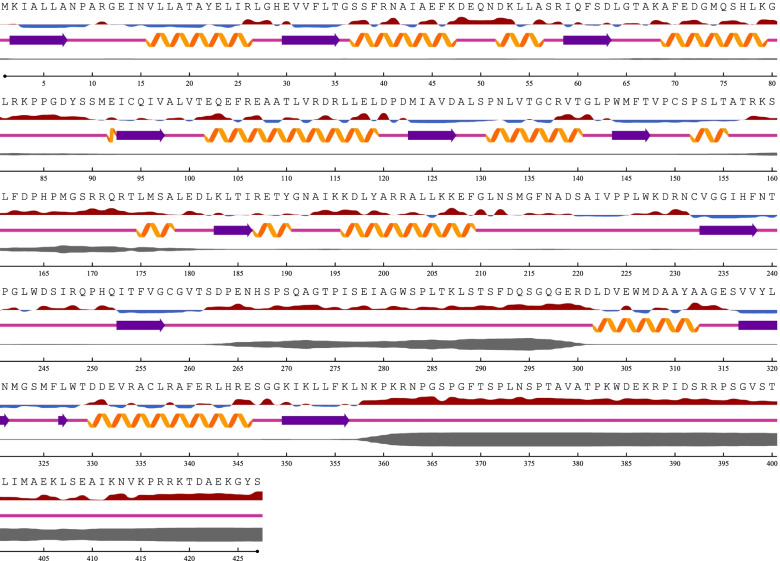
Secondary structure prediction for the PaEmt1p primary protein sequence for *M. antarcticus* reference strain T-34. Disordered residues are located from aa160 to aa180, from aa260 to aa300, and from aa358 to the end of the sequence

**Fig. 5:**
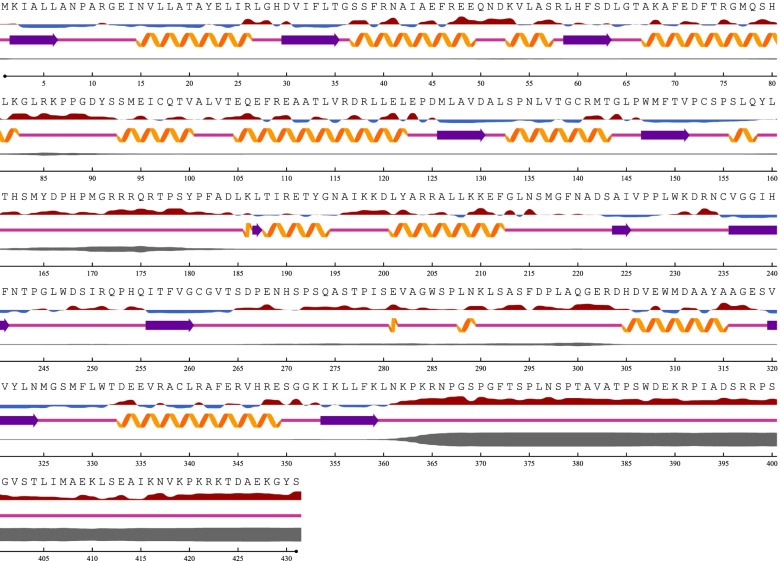
Secondary structure prediction for the PaEmt1p primary protein sequence of the representative *M. antarcticus* Trinidad strain. The comparative absence of disordered residues located at aa160 to aa180 and at aa260 to aa300; the longest length of disordered residues from aa357 to the end of the sequence has been retained

The predicted secondary structure of the reference sequence (strain T-34) and the representative Trinidad sequence was similar based on comparison of location and length of α-helices, β-pleated sheets and coils. Disordered residues toward the C-terminus were highlighted after aa358 to the end of the sequence. This region had the highest level of disorder in all three sequences. The least disorder, spanning the smallest region, was reported for aa160 to aa175 in both sequences. In the reference sequence of *M. antarcticus* strain T-34, disordered residues were located from aa160 to aa180, from aa260 to aa300, and from aa357 to the end of the sequence. In the Trinidad strain, disordered residues located from aa160 to aa180 and from aa260 to aa300 were notably absent; the longest length of disordered residues from aa357 to the end of the sequence was retained. Regions of disordered residues corresponded to exposed residues and not buried residues (Figs. 4 and 5).

### Phylogeny of Emt1 protein sequences

**Fig. 6:**
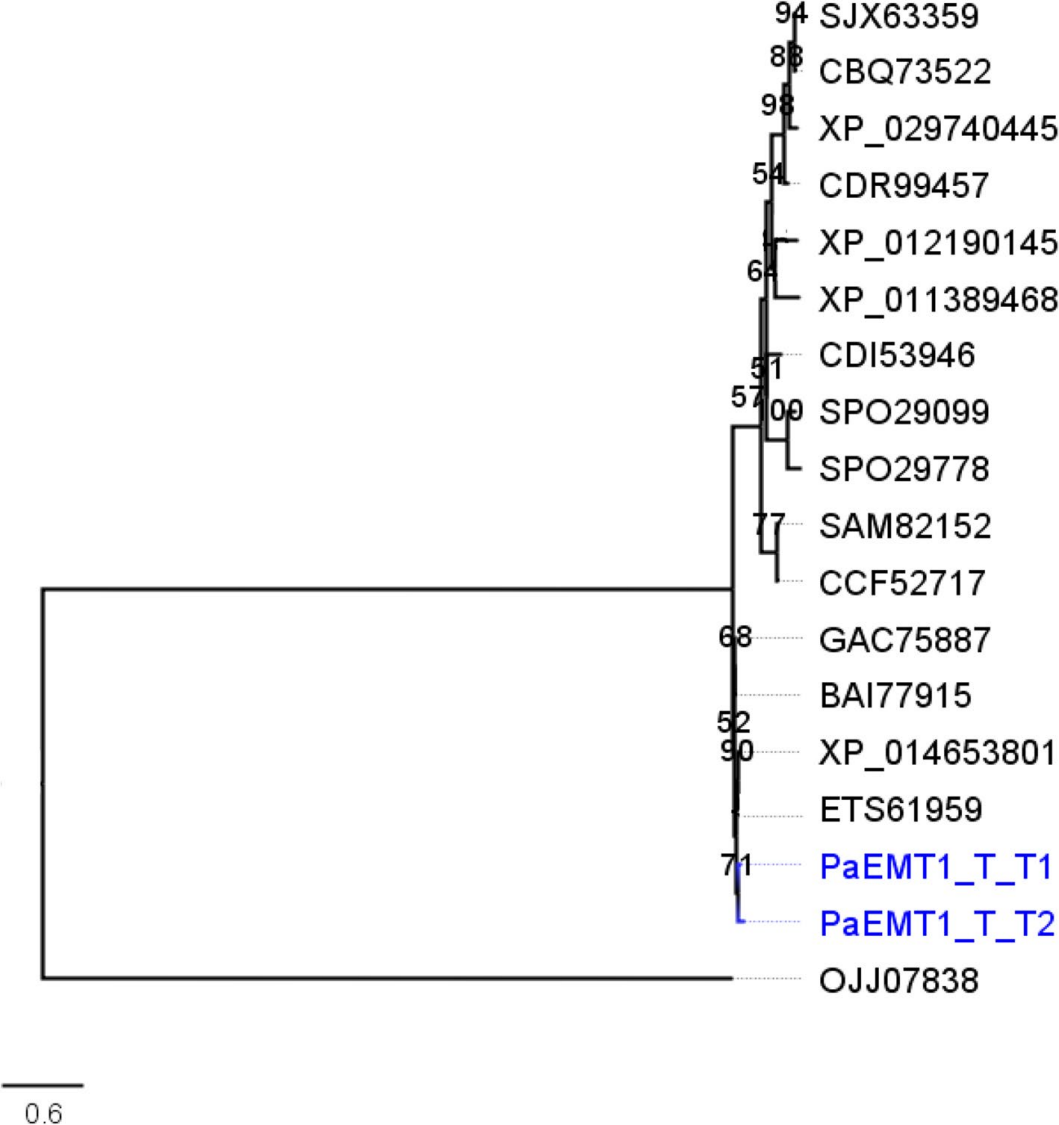
Phylogenetic analysis of deduced Emt1p sequences. The representative Trinidad strains are highlighted in blue; Emt1p sequences of the Trinidad strain are identical and as such, subsequent analyses of the other MEL genes were carried out on one consensus representative sequence

The ML algorithm was used to infer the phylogenetic relationships among the 18 Emt1p sequences each with 251 aa sites. The BIC best-fit model was the JTT + G4 model of aa substitution [54]. The ML 50% consensus tree is (Log-likelihood of consensus tree: − 3414.634125) after 1000 bootstrapped replicates is presented (Fig. 6). *Aspergillus* sp. was used as the outgroup.

Based on these results, it is proposed that the putative *PaEmt1* gene of the Trinidad strains encodes an erythritol/mannose transferase identical to that carried by strain T-34 reference sequence. The first step of MEL biosynthesis is condensation of mannosyl and erythritol catalyzed by the glycosyltransferase Emt1 which confirmed that the MEL BGC was present in the Trinidad isolates.

### Mac1 protein sequence analysis

There were three matches to *Moesziomyces*: GAC75889, ETS61961 and XP_014553798. The highest query coverage and identity percentage were obtained for GAC75889 reference sequence for *M. antarcticus* with 79% query coverage and 91.39% sequence identity match. There were lower value hits to *Ustilago* and *Sporisorium*.

No signal peptide sequences were present in the aligned aa sequences and there were no TM domains in the Trinidad sequences and none in the sequences of the four main related genera: *Moesziomyces*, *Sporisorium*, *Ustilago* and *Melanopsichium*. A conserved domain search confirmed the identity of the protein sequence as belonging to acyltransferase i.e. having transferase activity, transferring acyl groups and is involved in the proposed MEL biosynthetic route. *Mac1*- and *Mac2*-encoded proteins transfer short- and medium-chain fatty acids to positions R-2 and R-3 (Fig. 1). The last step, acetylation of deacetylated MEL at positions R-4 and R-6, is catalyzed by a single enzyme encoded by the *Mat1* gene (Fig. 1).

**Fig. 7:**
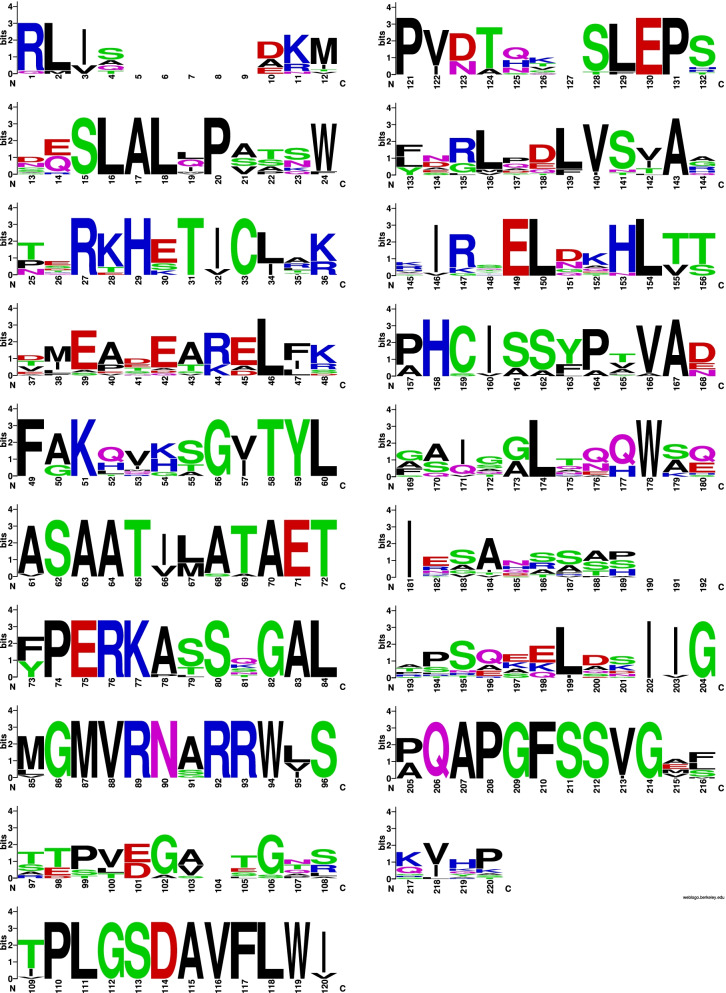
Mac1p sequence logo. The logo consists of stacks of symbols, one stack for each position in the amino acid sequence. The overall height of the stack indicates the sequence conservation at that position, while the height of symbols within the stack indicates the relative frequency of each amino acid at that position

There was a high level of variability in the aligned aa sequences (Fig. 7). Mac1p sequences were motif-rich compared to the other genes of the MEL gene cluster. Notably, consensus pattern: [STAGCN]-[RKH]-[LIVMAFY] in addition to a microbody C-terminal targeting signal “ARL” at aa550 to aa552 were detected in all compared Mac1p sequences.

A search for acyltransferase-specific motifs, ‘HXXXD’ and ‘DFGWG’, revealed that one motif, the ‘HXXXD’ as ‘HALAD’ was detected in the reference sequence (GAC75889) at position aa171 to aa181. This search was based on the entire acyltransferase protein sequence available in GenBank. In both higher plants and yeasts, the ‘HXXXD’ motif is highly conserved. The alignment used in this study, however, did not cover the entire sequence as 243 aa-sequences were available and therefore, included. The Trinidad Mac1p sequence was outside that of the reference strain T-34 sequence at both termini; thus, for the Trinidad sequences, the ‘HXXXD’ motif was found outside of the aligned sequences and consequently, outside of the deduced translated aa sequence.

### Phylogeny of Mac1 protein sequences

**Fig. 8:**
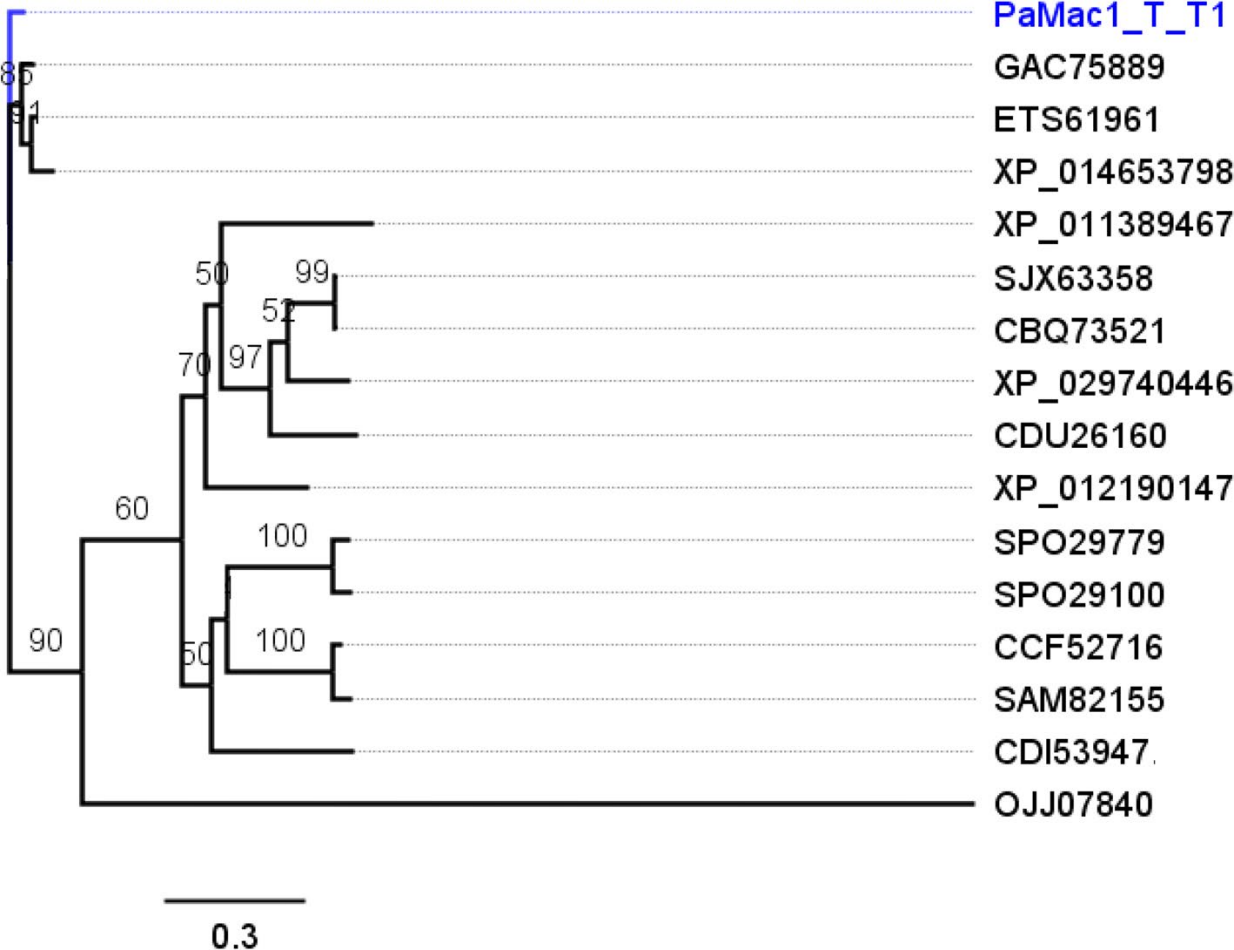
Phylogenetic analysis of Mac1p sequences. The representative Trinidad strain is highlighted in blue

The ML algorithm was used to infer the phylogenetic relationships among 16 aligned Mac1p sequences with 1000 replicates. The BIC best fit model was determined to be Gamma with 4 rate categories (LG + G4; LG) model of aa substitution [54]. The 50% consensus tree (Log-likelihood of consensus tree: − 3180.776804) is presented (Fig. 8). The outgroup was *Aspergillus* sp.

The Trinidad sequences clustered separately with three *Moesziomyces* sequences. There were two clusters that contained a mixed membership of *Ustilago, Pseudozyma* and *Sporisorium*. Based on these results, it is proposed that the putative *PaMac1* gene of the Trinidad strains encodes an acyltransferase which acylates the intermediate disaccharide mannosylerythritol in MEL biosynthesis which also confirmed that the MEL BGC was present in the Trinidad isolates.

### *Mac2* nucleotide and protein sequence analysis

For the *Mac2* nucleotide sequence of the Trinidad strain, analysis of both nucleotide and aa sequence was carried out because this was the only essential MEL gene with comparatively low sequence identities. There was one Blastn hit to *M. antarcticus* as a conserved hypothetical protein partial mRNA (XM_014804445) that is translated to an FAD-dependent sugar 1,4-lactone oxidase. The query coverage and identity similarity were comparatively low at 71 and 86.96%, respectively (E = 4e-126). Blastp comparison of the deduced translated aa sequence resulted in 61% query coverage with identity similarity of 96.58% (E = 9e-72) for *M*. *aphidis* strain DSM 70725 (ETS63302). Lower range matches were obtained for other members of *Ustilaginaceae* outside of the *Moesziomyces* genus.

**Fig. 9:**
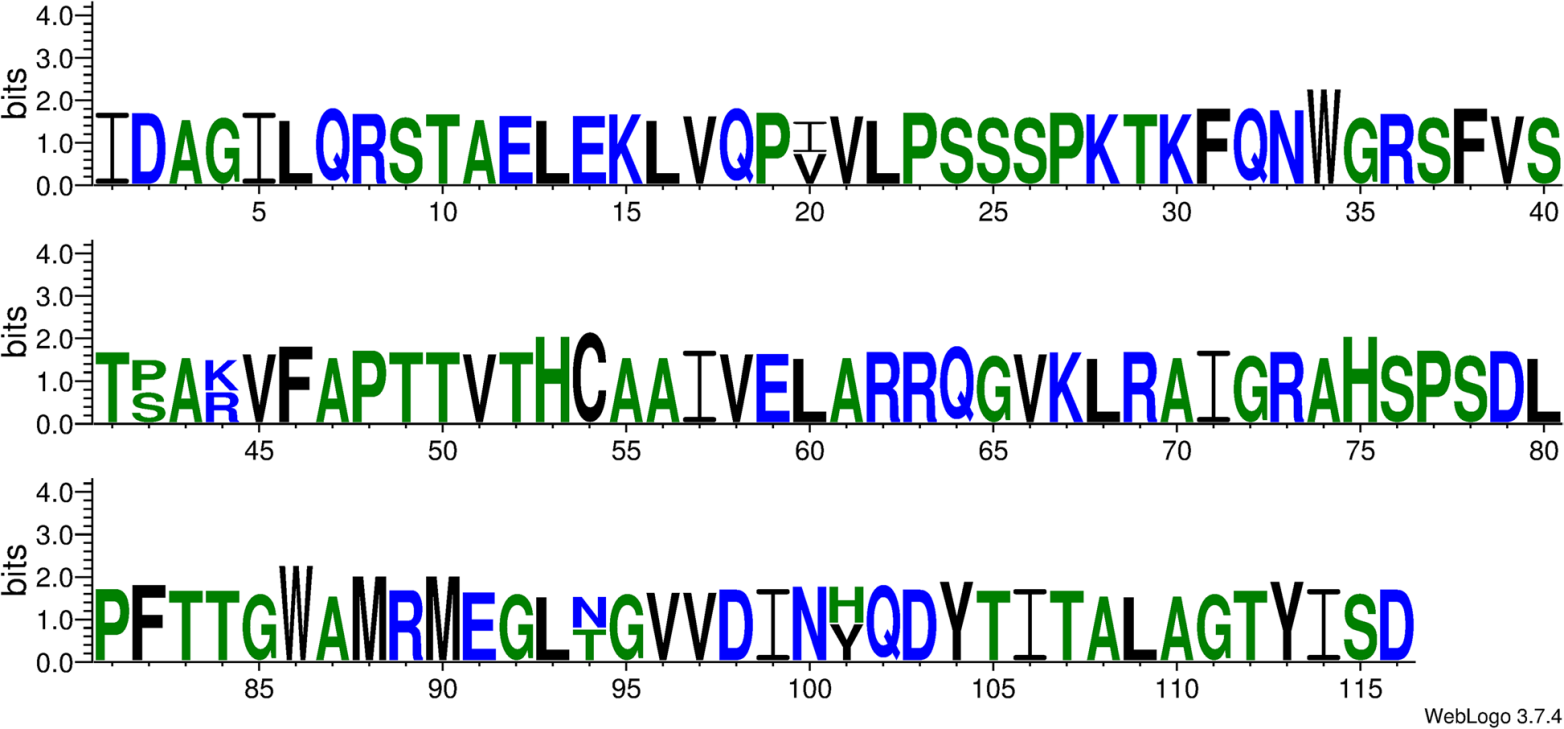
Mac2p sequence logo. The logo consists of stacks of symbols, one stack for each position in the amino acid sequence. The overall height of the stack indicates the sequence conservation at that position, while the height of symbols within the stack indicates the relative frequency of each amino acid at that position

The only motif detected was located between aa38 and aa116 which corresponded to an FAD-binding PCMH-type. This is in keeping with the identified enzyme which is FAD-dependent. There were no detected signal peptide sequences or TM domains present in the aligned aa sequences (Fig. 9).

### Phylogeny of Mac2 protein sequences

**Fig. 10:**
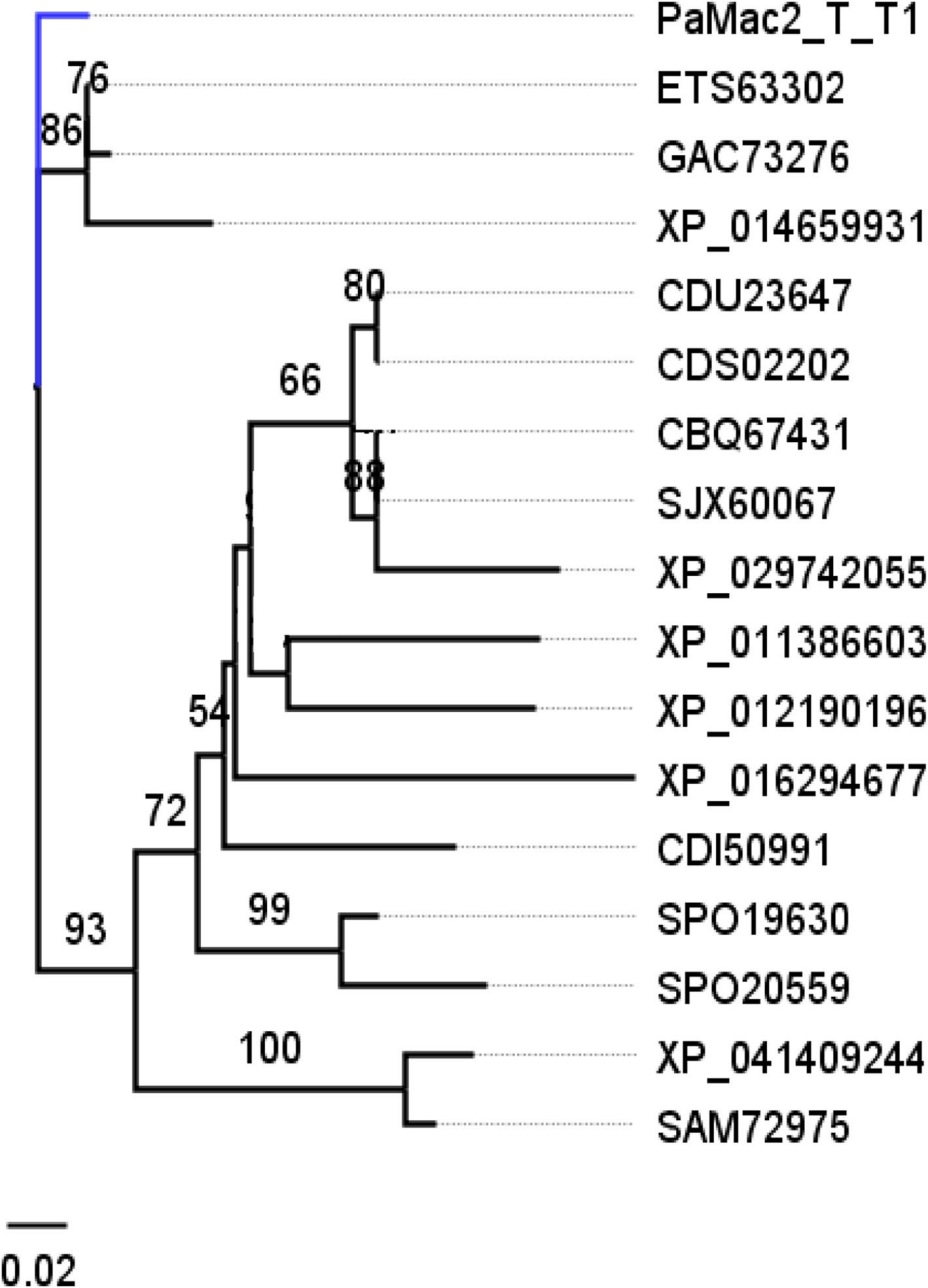
Phylogenetic analysis of Mac2p sequences. The representative Trinidad strain is highlighted in blue

The ML algorithm was used to infer the phylogenetic relationships among 17 aligned Mac2p sequences with 116 aa sites and 1000 replicates. The BIC best fit model was determined to be the JTT + G4 model of aa substitution. The 50% consensus tree (Log-likelihood of consensus tree: − 884.768978) is presented (Fig. 10). The outgroup was *Kalmanozyma brasiliensis.*

The Trinidad sequences clustered separately with the other three *Moesziomyces* sequences. Within this *Moesziomyces* clade, the Trinidad strain formed its own highly supported subclade. Based on these results, it is proposed that the putative *PaMac2* gene of the Trinidad strains likely encodes an acyltransferase which acylates the intermediate disaccharide mannosylerythritol in MEL biosynthesis to produce MEL with a different acylation pattern to that produced by Mac1p and which also confirmed that the MEL BGC was present in the Trinidad isolates.

### Mmf1 protein sequence analysis

**Fig. 11:**
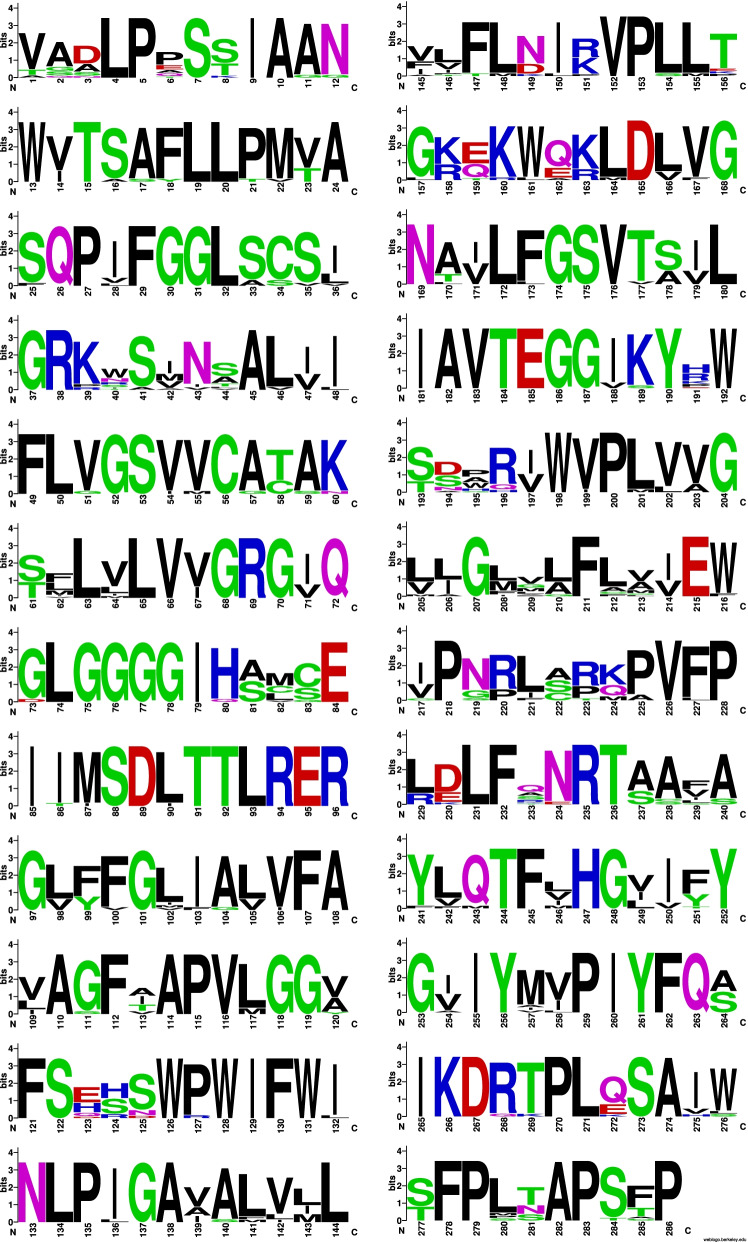
Mmf1p sequence logo. The logo consists of stacks of symbols, one stack for each position in the amino acid sequence. The overall height of the stack indicates the sequence conservation at that position, while the height of symbols within the stack indicates the relative frequency of each amino acid at that position

The highest match in terms of query coverage and identity percentage were obtained for three *Moesziomyces* sequences: ETS61962, XP_014653797, and GAC75890 in descending order of sequence similarity. Blastp query coverage was 99% but similarity ranged from 91.64 to 92.68%. Lower-value hits were obtained for *Ustilago* and *Sporisorium* sequences. The aligned protein sequences were variable with three or more aa substitutions at specific sites (Fig. 11). This would account for the sequence identity range obtained in the Blastp results.

The conserved motif detected in all aligned Mmf1p sequences was the major facilitator family of transporters which spanned aa1 to aa259. Signal peptide prediction based on neural networks and hidden Markov models (HMM) suggested a signal peptide at position aa27 and aa28 with ‘VAS-QP’ as the cleavage site for all sequences included in the alignment. This signal sequence was located closer to the N-terminus. Phobius also predicted several TM domains in the aligned protein sequences. There were seven putative TM helices located outside of the plasma membrane and seven TM helices found inside the plasma membrane. TMpred also confirmed that the predicted N-terminus was located inside the plasma membrane and the C-terminus was located outside of the plasma membrane and was, therefore, considered to be non-cytoplasmic. All Mmf1p sequences in the alignment had one predicted *N*-glycosylated site “NRTA” where the asparagine was considered to be glycosylated at aa234.

### Phylogeny of Mmf1 protein sequences

**Fig. 12:**
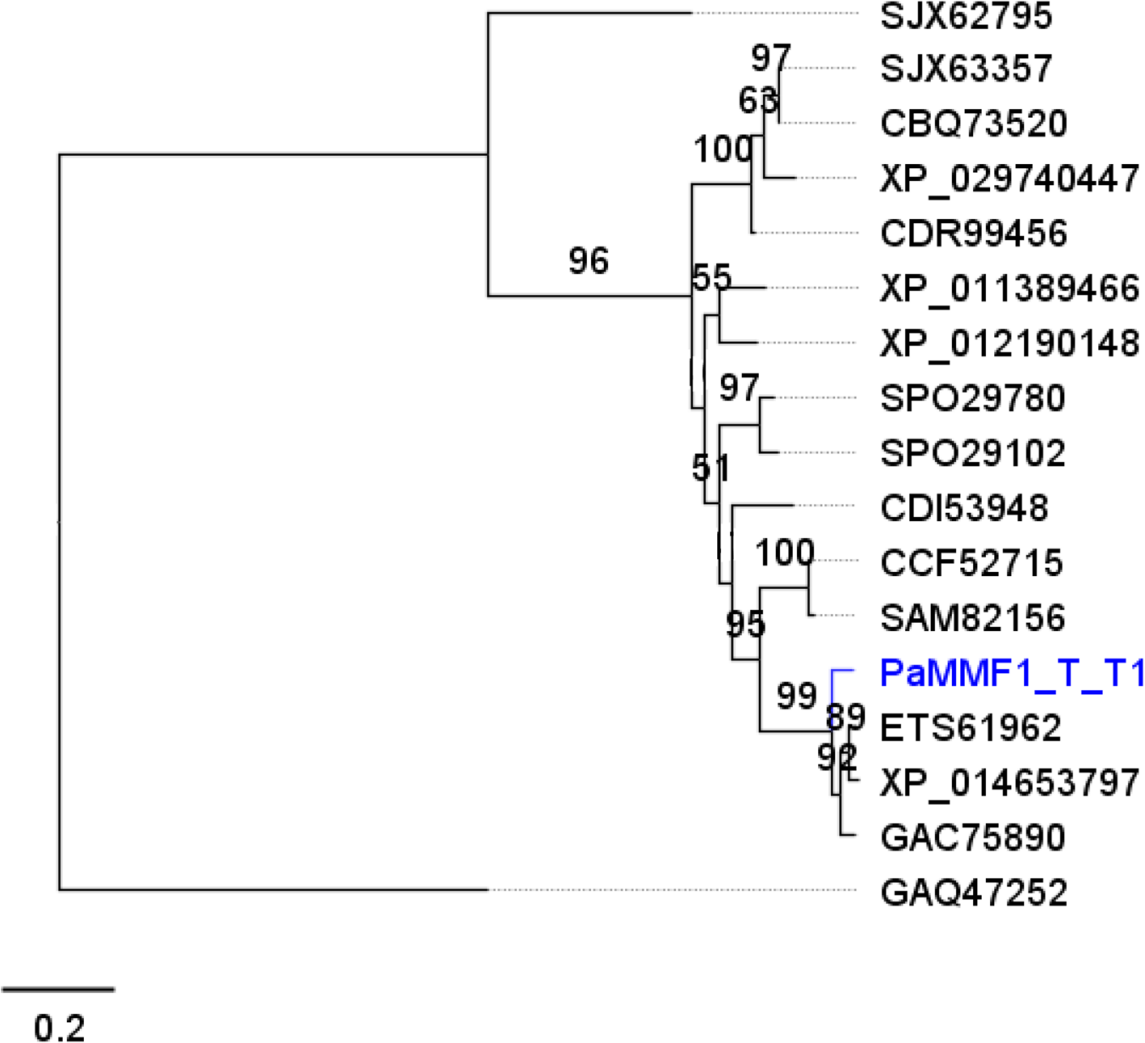
Phylogenetic analysis of Mmf1p sequences. The representative Trinidad strain is highlighted in blue

The ML algorithm was used to infer the phylogenetic relationships among 17 Mmf1p sequences with 287 aa sites. The BIC best-fit model was Gamma with 4 rate categories (LG + G4m); LG [54] model of aa substitution using *Aspergillus* sp. as the outgroup. The 50% consensus tree was constructed from 1000 bootstrap trees (Log-likelihood of consensus tree: − 3251.305052) (Fig. 12).

The representative Trinidad sequence clustered separately with the other three *Moesziomyces* sequences. Based on these results, it is proposed that the putative *PaMmf1* gene of the Trinidad strains encodes a secreted protein that belongs to the major facilitator transporter superfamily which is essential in the movement of a range of substrates across the plasma membrane. This data also confirmed that the MEL BGC was present in the Trinidad isolates.

### Lipase analysis

Both *LipA* and *LipB* genes (syn. Found on UniProt https://www.uniprot.org/uniprot/W3VKA4; Esther database http://bioweb.supagro.inra.fr/ESTHER/) were detected in the Trinidad strains through gene-targeted PCR amplification and sequencing of amplicons generated by the primer pairs designed in this study. Blastp comparisons resulted in 3GUU_A:35-244_*Candida antarctica* lipase A and *M. antarcticus* strain T-34 as most similar to the Trinidad sequence. A comparison of the aligned LipAp and LipBp sequences was then carried out with these two reference sequences against the representative LipAp and LipBp sequences of the *M. antarcticus* Trinidad strains. Signal peptide sequences were present in each of the aligned LipAp and LipBp sequences.

**Fig. 13:**
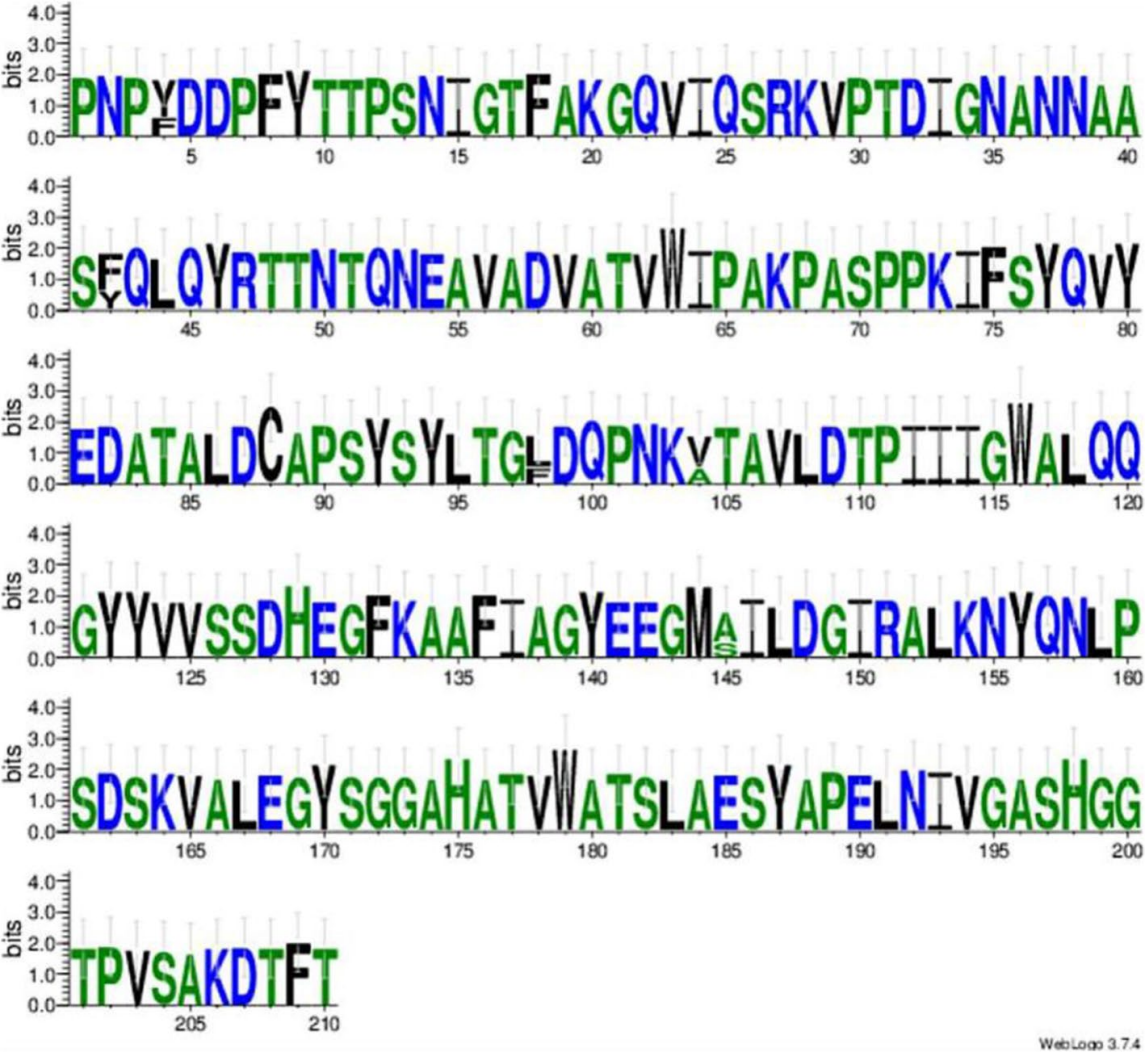
LipAp sequence logo. The logo consists of stacks of symbols, one stack for each position in the amino acid sequence. The overall height of the stack indicates the sequence conservation at that position, while the height of symbols within the stack indicates the relative frequency of each amino acid at that position

**Fig. 14:**
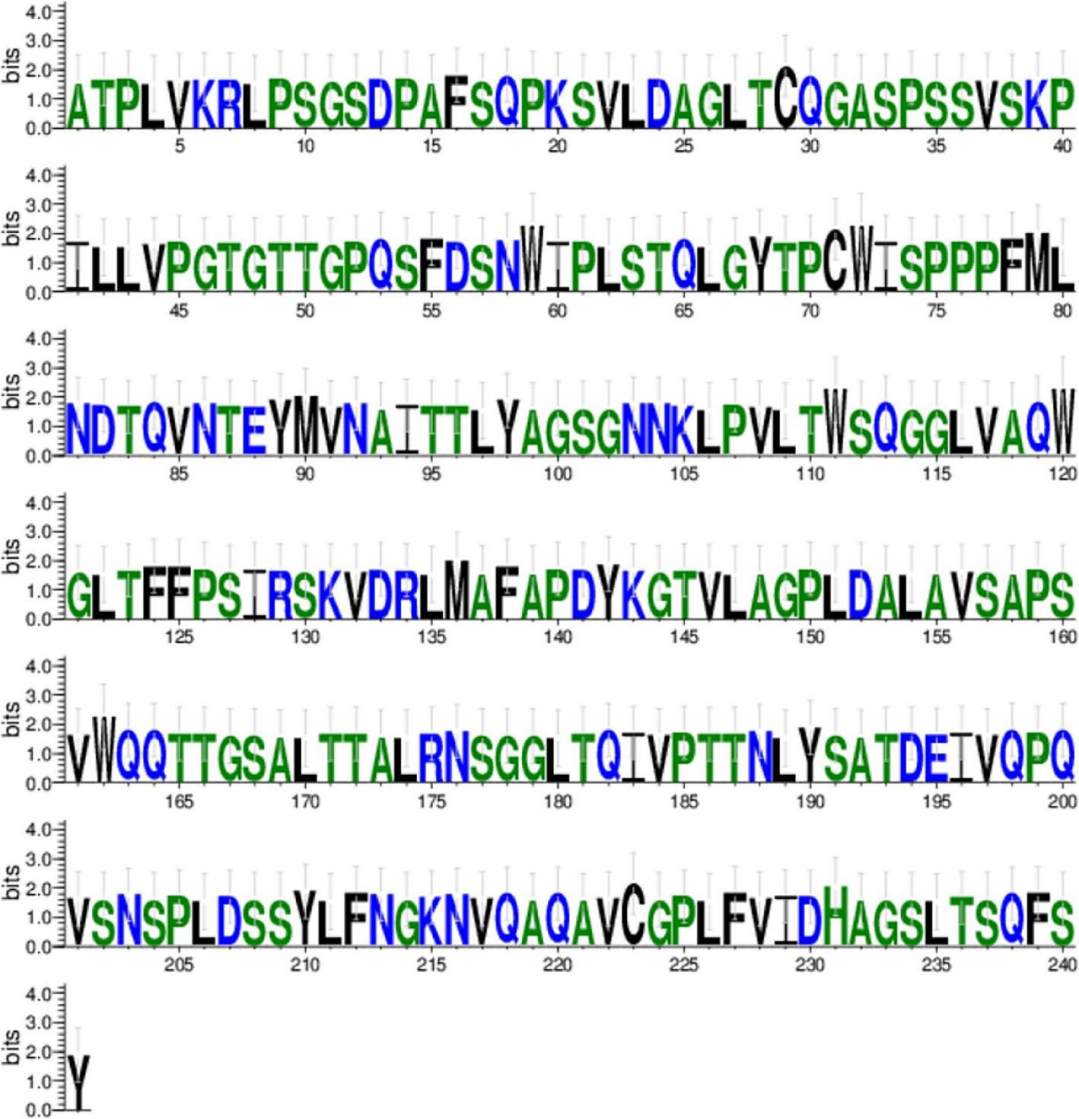
LipBp sequence logo. The logo consists of stacks of symbols, one stack for each position in the amino acid sequence. The overall height of the stack indicates the sequence conservation at that position, while the height of symbols within the stack indicates the relative frequency of each amino or nucleic acid at that position

Analysis of the LipAp and LipBp sequences indicated that LipAp was more variable than LipBp at four aa positions in the alignment and these four positions varied only in the Trinidad LipAp sequence; the aa substitutions were Y4F, L98F, V104A, and A145S (Fig. 13). PROVEAN prediction indicated that these four aa substitutions in the Trinidad sequence were neutral at a default threshold of − 2.5. This is in contrast to LipBp sequence analysis in which the LipBp sequence of reference strain T-34 was compared to that of the representative Trinidad strain. The LipBp sequences were 100% identical which indicated absolute consensus among the aa of this protein (Fig. 14). There were 17 records of LipBp sequence in the UniProt database (https://www.uniprot.org/uniprot/P41365) with 100% identity to each other and aa substitutions within this protein sequence generally led to reduced lipase activity in *M. antarcticus* depending on the type of mutation.

**Fig. 15:**
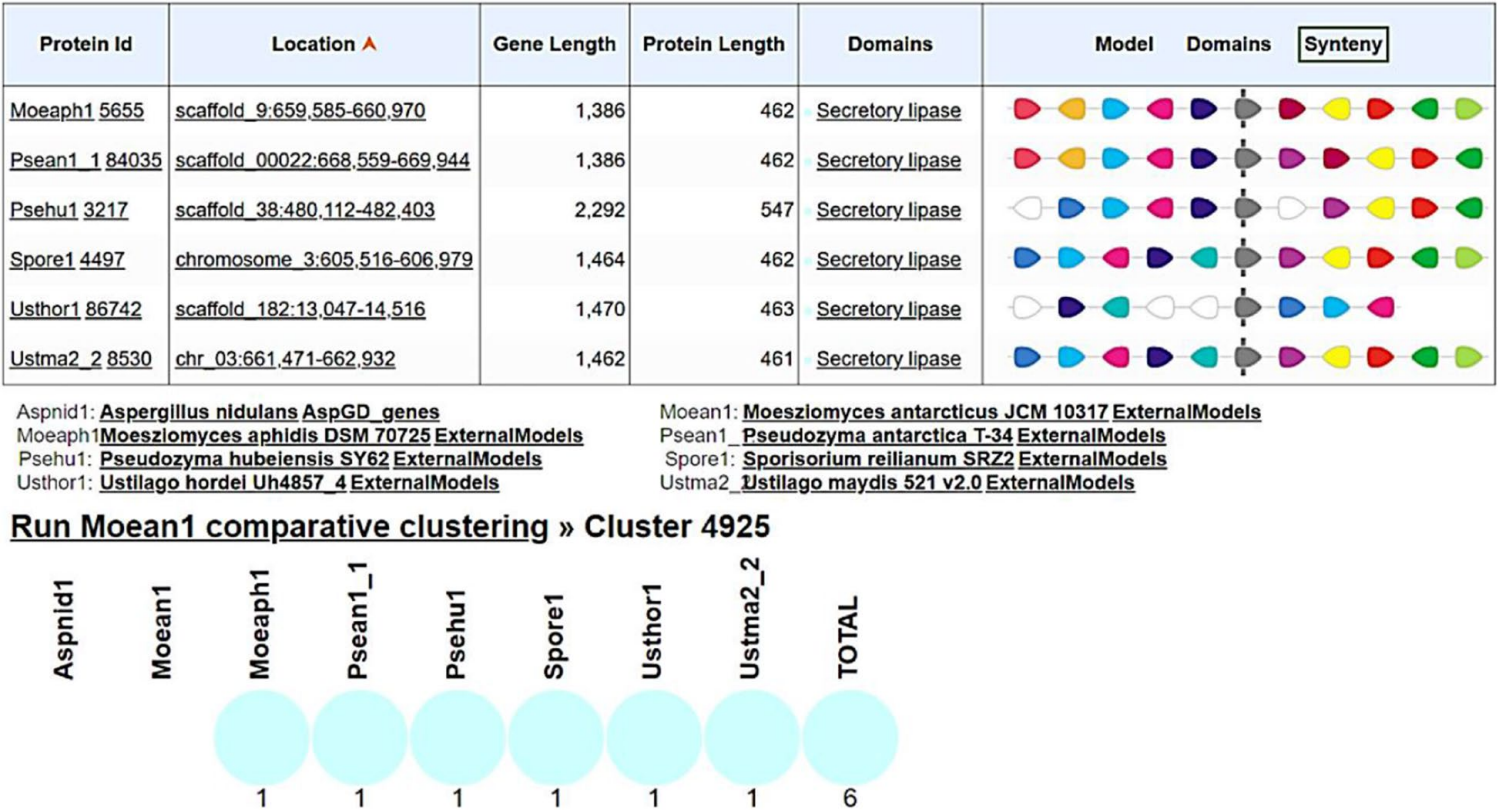
Genomic arrangement of lipase domain in *Moesziomyces* and related genera in MycoCosm

A comparison of secretory lipases among *Moesziomyces* and related genera in MycoCosm (https://mycocosm.jgi.doe.gov/) revealed a highly conserved genomic block arrangement (synteny for MCL cluster model #4925) (Fig. 15). There was rearrangement of adjacent domains which suggested that they were not critical to secretory lipase gene function.

### Comparative evolution of MEL BGC membership and *LipA*

**Fig. 16:**
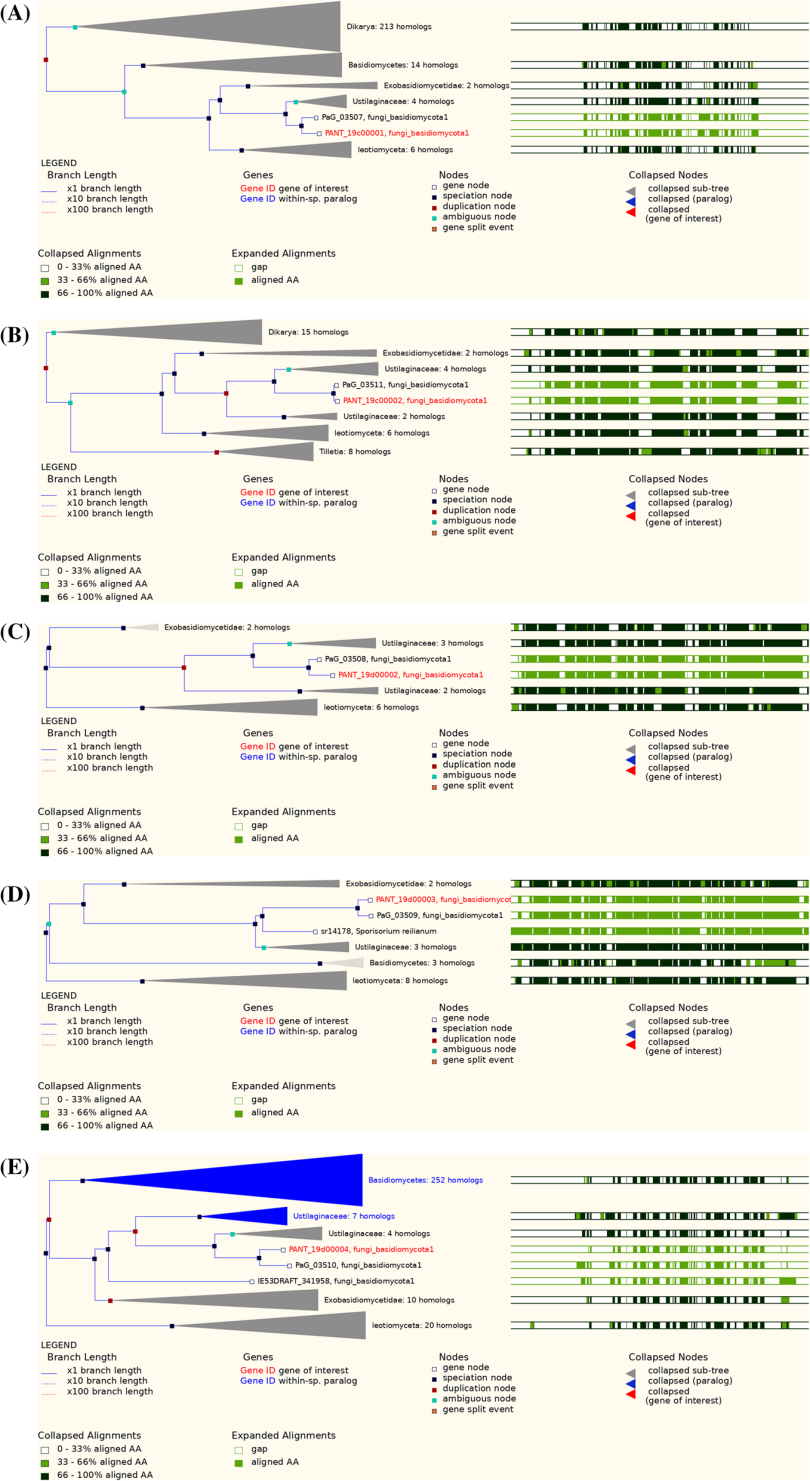
Comparative evolution of MEL BGC genes in EnsemblFungi. EnsemblFungi ID: **A** PANT_19c00001: *PaEmt1*; **B** PANT_19c00002: *PaMat1*; **C** PANT_19d000003: *PaMac1;* **D** PANT_ 19d00002: *PaMac2*; **E** PANT_19d00004: *PaMmf1*

The committed step in MEL biosynthesis is catalysed by the protein encoded by *PaEmt1* gene. Comparative evolution analysis of this gene suggested an early ancestral duplication event from which a number of speciation events occurred such that four homologues were detected within *Ustilaginaceae*. There were no proposed duplication events for *PaMac1*, only speciation events. For *PaMac2*, there was one detected duplication event that occurred later in evolutionary time compared to *PaEmt1*, and which occurred just before a speciation event that separated *Ustilaginaceae* into two groups, one with two homologues and another with three *PaMac2* gene homologues. There was one early ancestral duplication event proposed for *PaMat1*, followed by two later duplication events, one of which was followed by speciation events that led to two *Ustilaginaceae* groups - one with two homologues and the other with four homologues of this gene. Comparative evolution of *PaMmf1* gene suggested a duplication event immediately followed by speciation events where seven homologues existed for one group of *Ustilaginaceae* members and another group of *Ustilaginaceae* for which four homologues were detected. Comparative evolution of all genes suggested that *M. antarcticus* and *M. aphidis* reference strains were consistently separated from other members of *Ustilaginaceae*. Comparative evolution of MEL BGC genes is shown in Fig. 16.

**Fig. 17:**
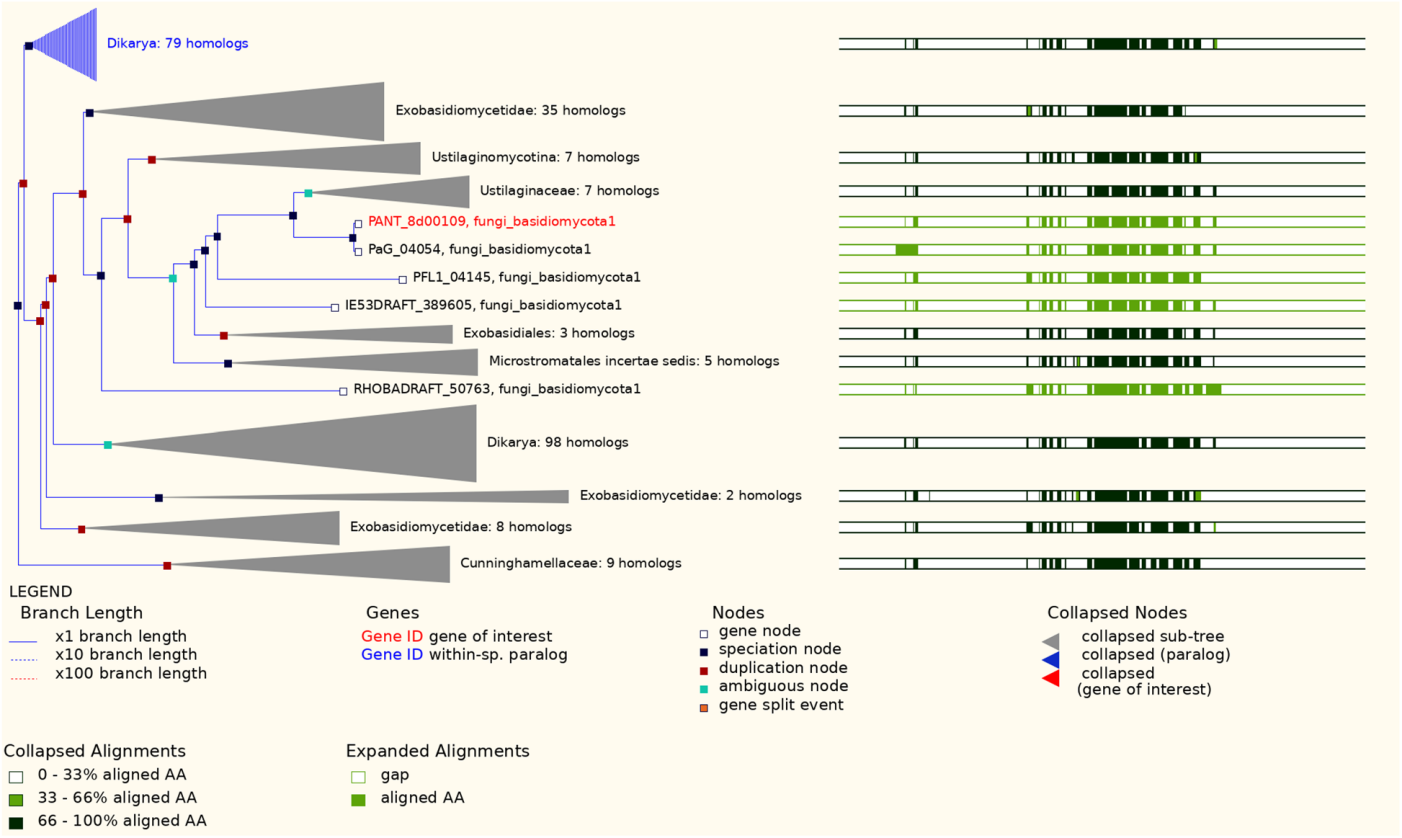
Comparative evolution of the *LipA* gene in EnsemblFungi

Comparative evolution of the *LipA* gene in EnsemblFungi indicated multiple duplication and speciation events for all homologues detected among all fungi in this database (Fig. 17). Specifically within *Ustilaginaceae*, there were four putative speciation events and two duplication events for two *Sporisorium reilianum* strains and two *M. brasiliensis* strains. A proposed speciation event, located at node ID 29170881, may have been defined by at least eight aa substitutions (A90T, E284D, A349T, G362Q, V388A, S389G, V418I and D505N) in *M. antarcticus* strain T-34/*M. aphidis* strain DSM70725, respectively (Wasabi viewer at node ID 29170888 in EnsemblFungi). Another proposed single speciation event, located at node ID 29170881, separated *M. antarcticus* strain T-34/*M. aphidis* strain DSM70725 from *U. maydis* strain 521*, U. hordei* (no strain provided)*, M. hubeiensis* strain SY62, two *Sporisorium reilianum* strains and two *M. brasiliensis* strains, and as a result, seven corresponding *LipA* gene homologues emerged for *Ustilaginaceae*.

### Bioassays

**Fig. 18:**
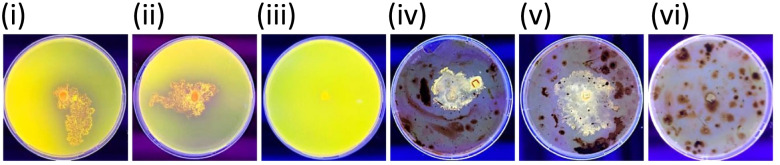
Lipase assays with Rhodamine 6G under UV light for (i and ii) isolates with olive oil as the carbon source, (iii) negative result with olive oil as the carbon source, (iv and v) isolates with crude oil as the carbon source and (vi) negative result with crude oil as the carbon source; yellow-to-orange-coloured fluorescence indicated the presence of extracellular lipase; no fluorescence indicated a negative result

Rhodamine 6G assays, conducted with olive oil and crude oil as carbon sources, both showed the presence of yellow-to-orange-coloured fluorescence under UV light indicating secreted lipase activity. Fluorescence obtained for each isolate and examples of positive and negative results are shown in Fig. 18.

## Discussion

In this study, three essential genes of the MEL BGC, *Emt1*, *Mac1* and *Mmf1*, and the dispensable *Mac2* gene, in addition to the *LipA* and *LipB* genes in the *M. antarcticus* Trinidad strains and in related strains in the *Ustilaginaceae* family were studied.

Phylogenetic inference based on protein sequence alignment from several related species are commonly used to determine relatedness among homologous sequences, and provides clues into the evolution of a protein family and the functional specificity of these protein members [55]. ITS rRNA sequence comparisons and phylogenetic analyses identified the Trinidad strains as *M. antarcticus*. The Trinidad strains shared the discrete and moderately-supported *M. antarcticus* clade with one *M. parantarctica* strain and two *M. parantarctica* strains. *M*. *parantarctica*, known to produce large amounts of MELs [28], was shown to be phylogenetically closely related to other known MEL-producers *M. antarcticus*, *M. rugulosa* and *M. aphidis* [56, 57]. The Trinidad strains were phylogenetically characterized by their ITS sequences which coincided with MEL production, a finding similar to Morita, et al. [28]. Hence, *M. antarcticus* formed a discrete subclade with *M. parantarctica* within the clade containing other well-known MEL-producers, *M. rugulosa* and *M. aphidis*. ITS phylogeny positioned *M. rugulosa* and *M. aphidis* with *M. antarcticus* sequences in a highly-supported clade with polytomic branching indicating that, for these species, the ITS sequences were too invariant for resolution at the inter-specific level.

Fungal genes that control primary metabolism are seldom clustered, however, gene clusters that regulate the production of secondary metabolites are common [29, 58]. The “selfish cluster” hypothesis purports that gene cluster organization facilitates the transfer of complete biosynthesis pathways upon lateral transfer [29]. Studies that assessed the function of *Emt1* using gene disruption methods found that it is essential for MEL biosynthesis regardless of the carbon source utilized [25, 43]. No signal peptides or TM domains were detected in the Emt1p of the Trinidad strains and for related genera of the *Ustilaginaceae* family (*Moesziomyces*, *Sporisorium*, *Ustilago*, and *Melanopsichium*) which suggested localization of this protein in the cytosol. Protein sequences without any evidence of signal peptides included in their sequences generally would not be glycosylated in vivo even though analytics show that they may contain potential glycosylation motifs.

A common sequon was present in most of the Emt1p sequences apart from the *Moesziomyces* sequences, having a glycosylation motif ‘NATK’ at aa195, and two *Ustilago* sequences had another motif ‘NSTS’ at aa293. The Trinidad strains had similar secondary structures to the *M. antarcticus* strain T-34. Disordered residues toward the C-terminus were highlighted after aa358 toward the end of the sequence, and was the highest level of disorder observed in all three sequences. For the three sequences, the smallest region of disorder was at aa160 to aa175. Conserved regions of disorder are associated with a range of biological activities. Comprehensive reviews on disordered proteins [59, 60] have highlighted the six functional classes of these activities [61]. In the first class, entropic chain classifiers, intrinsically disordered proteins or intrinsically disordered protein regions do not require ordered confirmation for their functioning. Intrinsically disordered protein regions can also have display site functions which provide conformational flexibility that allows access to the protein backbone by posttranslational modification enzymes which facilitates glycosylation. Such functional classification may hold true for the intrinsically disordered protein regions present in the Emt1p sequences under study.

The second step in the assembly of the MELs involves acylation of mannosylerythritol which is catalyzed by two acyltransferases encoded by *Mac1* and *Mac2* genes. *Mac2*-encoded acyltransferase may be dispensable for catalysis since it was found to lack the required aspartate residue of the nearly invariable acyltransferase motif ‘HXXXD’ that is part of the enzyme’s active centre [29, 62]. The ‘DFGWG’ motif is found in the C-terminal domain, and is important to maintaining the structural geometry of the enzyme [63]. Phylogenetic analysis placed the putative aa sequence encoded by *Mac1* of the Trinidad strains with the highest similarity to that of *M. antarcticus* strain T-34, and to two other *Moesziomyces* sequences. More distantly related taxa had a mixed membership with related genera of the *Ustilaginaceae* family (*Pseudozyma*, *Sporisorium* and *Ustilago*).

Alignment of the Mac1p sequences exhibited high levels of variability, however, these sequences were motif-rich in relation to other genes in the MEL cluster. A clear consensus pattern [STAGCN]-[RKH]-[LIVMAFY] and a C-terminal targeting signal “ARL” at aa550 to aa552 was detected in all Mac1p sequences. This confirmed the localization of this enzyme to the peroxisomes for β-oxidation [64]. Peroxisomes participate in the synthesis of various secondary metabolites in fungi [64], and *M. antarctica* was found to contain many genes for peroxisomal β-oxidation [42].

The enzymes responsible for MEL biosynthesis are compartmentalized to enable production of differently acylated MELs, and to allow for simultaneous assembly of different glycolipids in a single cell requiring the same precursor [65]. Mat1p and Mac1p of the Trinidad strains were confirmed to be localized in peroxisomes. In contrast, Emt1p glycosylation occurs in the cytosol as confirmed by the absence of these targeting signals in the Trinidad strains. Mat1p, which is not essential for MEL production and is located in the plasma membrane, is responsible for the production of three acetylated variants, MEL-A,-B,-C. Once MELs are produced, they must be translocated to the cell membrane for extracellular deposition. Membrane-bound transporter, Mmf1p, a member of the major facilitator superfamily, exports MEL-A,-B,-C,-D variants out of the cell. The *Mmf1* gene sequence in the Trinidad strains was highly similar to that of *M. antarcticus* and *M. aphidis*. Lower protein sequence similarity was obtained for related genera of the *Ustilaginaceae* family (*Sporisorium* and *Ustilago*). As such, phylogenetic inference of the Mmf1p sequences placed the Trinidad strain with other strains of *M. antarcticus* and *M*. *aphidis*. In addition, the characteristic features of signal peptide and TM regions required for secretion of MELs confirmed the localization of the protein to N-terminus inside the plasma membrane and the C-terminus outside the plasma membrane.

*M. antarctica* is an excellent producer of industrial lipases [66]. Both *LipA* and *LipB* genes in the Trinidad strains were identified using gene-specific primers. Blastp indicated the top hit to lipase A of *M. antarcticus* strain T-34. Synonymous aa variations were found in LipAp. The LipBp sequences were highly conserved. *LipA* genes remain conserved in the *M. antarcticus* Trinidad strains. These putative secretory lipases in *Moesziomyces* and related genera originated from a common ancestor.

In this study, molecular characterization and functional analyses were conducted as opposed to common screening methods for the detection of biosurfactants [67, 68]. Though commonly used, basic sampling and isolation methods based on surface/interfacial activity (e.g. oil spreading assay, drop collapse assay) detect presence/absence of biosurfactants, and give no insight into the molecular aspects that enable their secretion [68]. Specialty media such as CTAB [69] and hemolytic assays are often used [68]. CTAB assays are semi-quantitative; CTAB is harmful and inhibits the growth of some microbes. CTAB assays were performed in this study according to Siegmund and Wagner [69] but, the results were inconclusive as uncharacteristic halos were formed (data not presented). Hemolytic assays are unreliable due to false negative and /or positive results. For example, there can be biosurfactant activity without hemolytic activity and microbes positive for hemolytic activity can be negative for biosurfactant production. In addition, biosurfactants that diffuse poorly in agar may not be able to lyse blood cells [70, 71].

The reference *M. antarctica* strain was initially isolated from the bottom of a lake in Antarctica. Morita, et al. [43] proposed that MELs functionality may contribute to low-temperature and freezing tolerance for survival under these extreme conditions. The secretion of lipases in Antarctic fungi can be associated with their need for maintenance of cell membrane fluidity in extreme cold conditions to survive [72, 73]. The Trinidad strains demonstrated tolerance to extreme crude oil pollution and MEL functionality may contribute to survival under these atypical conditions. *Moesziomyces* species have rarely been described as capable of crude oil detoxification. *M*. *aphidis* was shown to be efficient in degrading tetradecane [74], *Moesziomyces* sp. degraded diesel fuel [75], and *P. antarctica* can convert n-alkanes (C_12_ to C_18_) into MELs [76].

This is the first report of a gene-targeted approach to identify and to explain the ability of *M. antarcticus* Trinidad strains to degrade crude oil for remediation of polluted environments. *M. antarcticus* possesses more genes responsible for fatty acid transport and metabolism compared with *S. cerevisiae* [28, 44]. These results, in addition to those of other studies [28, 42], indicated that *M. antarcticus* have an adaptive advantage to surviving oleaginous conditions which explains the survival of the Trinidad strains inhabiting terrestrial sites chronically contaminated with crude oil. The conditions for activation and synthesis of these and other lipid metabolism-associated enzymes in tropical strains are expected to differ from those *M. antarcticus* strains isolated from sub-zero temperatures. Further studies of these and other secondary metabolite biosynthesis pathways will reveal how these pathways can be manipulated for industrial purposes [77, 78].

## Conclusions

This work explored the mechanistic and evolutionary underpinnings of tolerance and adaptation to oil-polluted terrestrial environments by specific *M. antarcticus* yeast strains indigenous to Trinidad. The experimental design included a novel gene-targeted approach for detecting and analysing essential genes of the MEL BGC and *LipA* and *LipB* genes. Sequence analyses indicated that these strains do not necessarily work to protect the genome but instead, the focus has been to protect the function of specific proteins that enable survival of these Trinidad *M. antarcticus* strains. It was found that the intrinsic ability of these strains to outcompete and survive in anoxic, low-nutrient terrestrial environments is necessarily mechanistically- and evolutionarily-linked. Bioremediation capability may also be related to protein domains that were intrinsically disordered which have known or suspected links to abiotic stress tolerance. These findings suggest that the Trinidad strains should be explored as promising candidates for the commercial production of MELs and lipase enzymes. As such, whole genome sequence analysis is currently being conducted for these strains.

## Methods

### Materials

Crude oil was obtained from CARIRI - Caribbean Industrial (St. Augustine, Trinidad and Tobago). Potato dextrose agar plates (PDA), yeast malt agar (YM), basal salts medium (BSM), Bushnell-Haas agar (BHA) composed per liter of: MgSO_4_ (0.2 g), CaCl_2_ (0.02 g), KH_2_PO_4_ (1.0 g) K_2_HPO_4_ (1.0 g), NH_4_NO_3_ (1.0 g), FeCl_3_ (0.05 g), and agar (20.0 g), and nutrient agar (NA) media were obtained from HiMedia Laboratories LLC (West Chester, PA, USA). Streptomycin, tetracycline, glycerol, olive oil, and Rhodamine 6G solution were obtained from Sigma-Aldrich (St. Louis, MO, USA). Maxwell® 16 Cell DNA Purification kits used for DNA extraction, and GoTaq® Green Master Mix and Nuclease-Free water for PCR were obtained from Promega (Madison, WI, USA). Primers were from Integrated DNA Technologies (Coralville, IA, USA). The Thermal Cycler 2720 to perform PCR was from Thermo Scientific (USA), and the MiniBIS Pro System to view PCR products was a DNR Bio Imaging System (Neve Yamin, Israel).

### Site description and sample collection

**Fig. 19:**
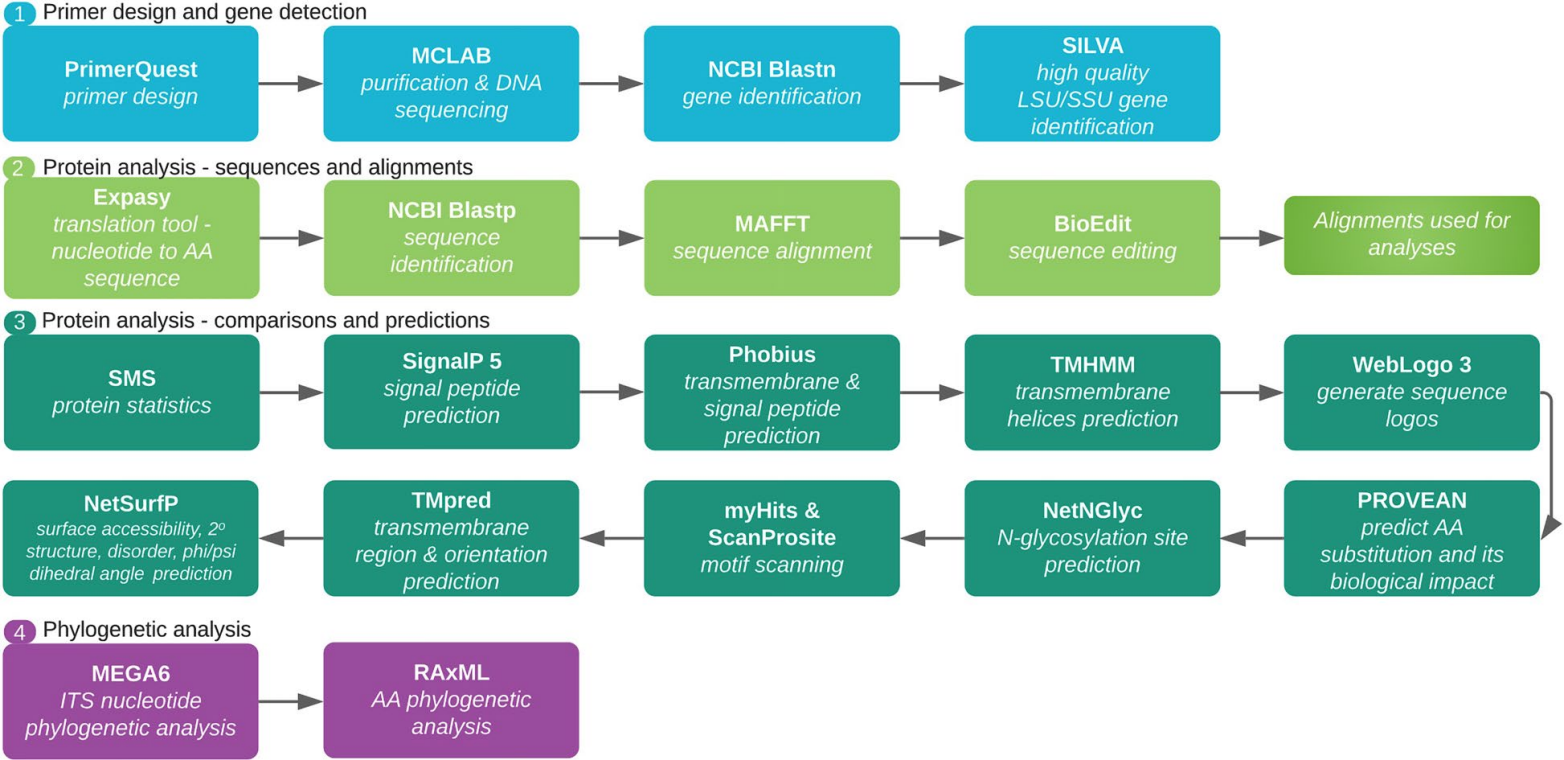
Bioinformatics pipeline

The sites under study included Vance River (10°12′06″N, 61°37′52″W) located in Siparia Region and the Marac Mud Volcano (10.0774° N, 61.3531° W) located in La Lune, Moruga (Fig. 19). Both sites are located in South Trinidad and are chronically polluted with crude oil [79, 80]. Such long-term exposure presents an opportunity to study microbes that have adapted to extreme conditions. An in-depth description of these sites can be found in Ramdass and Rampersad [81].

Contaminated soil samples were collected from five sites along Vance River, and included samples from oil contaminated sediments surrounding leaking pipelines, a natural oil seep where the surrounding land was heavily impregnated with oil and sites along the river containing oil runoff accumulated in sediment (Fig. 19). Samples of mud mixed with oil were taken from the volcano site at Marac Mud Volcano, and included samples from inside the vents, the edge of the vents, and the edge of the vegetation margin (Fig. 19). Approximately 500 g soil samples were collected from these sites at a depth of 10 cm into the subsurface using a stainless steel shovel. Debris (e.g. pebbles, leaves and twigs) were removed from the samples prior to placing into sterile Whirl-Pak bags on ice for transportation to the laboratory. The samples were stored at 4 °C for next-day processing.

### Isolate recovery and enrichment

Fungal isolation was performed as previously described [81]. Briefly, each hydrocarbon contaminated soil sample was serially diluted and aliquots spread over the surface of PDA supplemented with 50 mg/L each of streptomycin and tetracycline. Morphologically different colonies were then selected. The isolates were maintained on PDA plates and in 1.5 mL centrifuge tubes in sterile distilled water at 4 °C for short term storage, and in 15% glycerol at − 20 °C for long term storage. Stock cultures were cultivated at 25 ± 2 °C on YM containing 1% glucose, 0.5% peptone, 0.3% yeast extract, 0.3% malt extract and 1.5% agar. They were stored at 4 °C and renewed every 3 weeks.

For the isolation of crude oil-degrading microbes, biosurfactant- and lipase-producing microbes, enrichment cultures utilizing hydrophobic compounds as the sole carbon source were applied [68]. Mud and oil composite (~ 20 g) into a 500 mL conical flask with 500 mL BSM [82] supplemented with 2% (v/v) crude oil. The cultures were incubated for 2 days at 37 ± 2 °C with shaking (200 rpm). Following incubation, 1 mL of the mixture was transferred into another flask containing crude oil medium and maintained under the same conditions. The mixture was centrifuged (3000 rpm) after incubation, and the pellet was re-suspended in sterile phosphate buffer pH 7.2. Serial dilutions (10^− 1^ to 10^− 5^) were prepared and 100 μL aliquots were plated on 2% oil-amended PDA media and incubated as previously described.

### DNA extraction, PCR and sequencing

Isolates grown on oil-amended media were selected and identified based on partial sequence comparisons of the internally transcribed spacer region, ITS1–5.8S-ITS2 rDNA array (ITS4/5) [83]. LSU [84, 85] and SSU rRNA were also targeted (https://sites.duke.edu/vilgalyslab/rdna_primers_for_fungi/; Vilgalys Lab, Duke University). The LSU region contains two hypervariable domains, D1 and D2 [86], which when combined with ITS sequence data, can be valuable for fungal species identification [87].

Total genomic DNA from fungal isolates was extracted using the MoBio PowerSoil DNA extraction kit according to the manufacturer’s instructions. DNA extracts were diluted 1:4 and this served as the working DNA concentration for PCR amplification. PCR reactions were carried out on Thermal Cycler 2720. The PCR mixture (25 μL total volume) contained 12.5 μL of GoTaq Green Master Mix, 0.5 μL (10 μM) of each primer, 6.5 μL of Nuclease-Free water and 5 μL of DNA template. PCR reaction conditions consisted of an initial denaturation of 5 min at 94 °C followed by 35 cycles of 1 min of denaturation at 94 °C, 1 min of annealing at 55 °C, 1 min primer extension at 72 °C, followed by a final extension of 5 min at 72 °C. PCR products were examined on 1.5% agarose gels using the MiniBIS Pro System, and the amplicons were sent for purification and sequencing (MCLAB, San Francisco, CA, USA).

### Identification of isolates

Sequences of each amplified gene region were compared to sequences deposited in GenBank using BLASTn algorithm specifically against the ITS1–5.8 s-ITS2 rDNA TYPE SEQUENCE database. Confirmation of ITS sequence identity (GenBank Accession No. MZ143989-representative ITS sequence) was then carried out based on comparative BLASTn. The SILVA server (https://www.arb-silva.de/; high quality ribosomal RNA database) was used for molecular identification at the intermediate taxonomic level using LSU and SSU gene sequences. Sequences were aligned in MAFFT (https://www.ebi.ac.uk/Tools/msa/mafft/) [88] and edited in BioEdit [89].

To confirm sequence identity, phylogenetic inference was carried out using the ML algorithm with 1000 bootstrapped replications in MEGA [90]. Reference sequences were mined from the top matches in GenBank and these were included in the final dataset (Supplementary Table S1). Sequences were aligned in MAFFT (https://www.ebi.ac.uk/Tools/msa/mafft/) [88] and edited in BioEdit [89].

### Detection of MEL BGC

Gene-specific primers were designed to target three key genes in the MEL BGC: *PaEmt1*, *PaMac1*, *PaMac2* and *PaMmf1*. The reference protein sequences of type strain T-34 of *M. antarcticus* (GenBank GAC75887) was used in primer design. Primers were designed using IDT primer quest software (https://www.idtdna.com/PrimerQuest/Home/Index) and produced by IDT-Integrated DNA Technologies Inc. (Table 1). The PCR mixture (25 μL total volume) contained 12.5 μL of GoTaq® Green Master Mix, 0.5 μL (10 μM) of each primer, 6.5 μL of Nuclease-Free water and 5 μL of DNA template. PCR reaction conditions consisted of an initial denaturation of 5 min at 94 °C followed by 35 cycles of 1 min of denaturation at 94 °C, 1 min of annealing at the calculated Tm of each primer pair, 1 min primer extension at 72 °C, followed by a final extension of 5 min at 72 °C. PCR products were examined on 1.5% agarose gels using the MiniBIS Pro System, and the amplicons were sent for purification and sequencing (MCLAB, San Francisco, CA, USA).

**Table 1:** Primers designed in this study for detection of MEL biosynthetic gene cluster

Gene target	Primer orientation	Primer Sequence (5′-3′)	T_**m**_^**a**^/°C	Amplicon size/bp
PaEmt1	Forward Primer	TGTCTGCGCTCGaaAGTaaG	62.194	848
	Reverse Primer	GGAGAGCATaaACTGCGAGTAG	62.194	
PaMac1	Forward Primer	GCATCTCGGAGCTGTACaaT	61.919	996
	Reverse Primer	CGTGGaaCTTGGCATCaaAC	61.872	
PaMac2	Forward Primer	ACTTGCCCTTTGGTCTGTT	61.999	685
	Reverse Primer	ATTATCCGCCGCCTTGATT	61.986	
PaMmf1	Forward Primer	GCTGATGCTGATTGCCTTTC	61.803	994
	Reverse Primer	CCATCCGAGGaaGATGAGATTT	61.823	
PaLipA	Forward Primer	GCGCTCaaGaaCTACCAGaa	62.016	666
	Reverse Primer	GTCGaaGGCTTGCTTGATaaAC	61.941	
PaLipB	Forward Primer	AGCCACTCCTTTGGTGaaG	61.847	724
	Reverse Primer	GTaaGAGaaCTGCGAGGTGAG	61.804	

^a^ T_m_: primer annealing temperature

The nucleotide sequences of each amplicon were translated into deduced aa sequences using the Expasy translation tool (https://web.expasy.org/translate/) and the correct reading frame was confirmed. The identities of the deduced aa sequences were verified by BLASTp analysis.

### Bioinformatics analyses

Figure 19 outlines the bioinformatics workflow used in this study.

### Emt1, Mac1, Mac2, Mmf1 and LipA/B amino acid sequence analyses

The aa sequences of the MEL genes of the *M. antarcticus* strains were analysed to assess protein functionality of these genes and their associated gene products. Basic protein sequence statistics of the alignments were compared using the Sequence Manipulation Suite (SMS) server (https://www.bioinformatics.org/sms2/protein_stats.html) [91]. SignalP 5.0 (http://www.cbs.dtu.dk/services/SignalP/) was used in the detection of potential signal peptides using deep neural networks [92]. Phobius (https://phobius.sbc.su.se/) was used to determine whether there were TM domains in the protein sequences [93]. On the DTU heath Tech domain (https://services.healthtech.dtu.dk/) TMHMM v. 2.0 (https://services.healthtech.dtu.dk/service.php?TMHMM-2.0) was also used in the prediction of TM helices in proteins using hidden f model once there is an indication of such according to the Phobius results [94]. TMHMM has been rated best in an independent comparison of programs for prediction of TM helices [95].

Weblogo (https://weblogo.berkeley.edu/logo.cgi) was used to create a sequence logo for each protein sequence [96, 97]. In general, a sequence logo provides a richer and more precise description of, for example, a binding site, than would a consensus sequence. The PROVEAN software tool (http://provean.jcvi.org/index.php) was used to predict if aa substitution has an impact on the biological function of a protein. NetNGlyc (http://www.cbs.dtu.dk/services/NetNGlyc/) was used to predict potential *N*-glycosylation sites in the sequences through a search for Asn-Xaa-Ser/Thr sequons [98]. Not all asparagines in this consensus tripeptide are glycosylated as folding of the protein determines whether the Asp in the sequon is glycosylated. myHits Motif Scan (https://myhits.isb-sib.ch/cgi-bin/motif_scan) and ScanProsite (https://prosite.expasy.org/scanprosite/) were used to detect sequence specific motifs depending on the results of previous analysis on signal peptide and TM prediction. Depending on these results and the protein sequence under study, TMpred (https://embnet.vital-it.ch/software/TMPRED_form.html) was used to predict the membrane-spanning regions and their orientation relative to the plasma membrane and cytosol. These tools were included in the package of protein sequence analysis available in the Expasy Bioinformatics Resource Portal (https://www.expasy.org/).

On the DTU heath Tech domain (https://services.healthtech.dtu.dk/) [94] NetSurfP-2.0 (https://services.healthtech.dtu.dk/service.php?NetSurfP-2.0) was used to predict the surface accessibility, secondary structure, disorder, and phi/psi dihedral angles of aa in each deduced protein sequence. RAxML (Randomized Axelerated Maximum Likelihood) (https://raxml-ng.vital-it.ch/#/) was used to infer phylogenetic relationships for each protein using the ML algorithm [99, 100]. Reference sequences were mined from the top matches in GenBank and these were included in the final dataset (Supplementary Tables S2 to S5). Sequences were aligned in MAFFT (https://www.ebi.ac.uk/Tools/msa/mafft/) [88] and edited in BioEdit [89]. Phylogenetic trees were edited in FigTree 1.4.4 (http://tree.bio.ed.ac.uk/software/figtree/). A comparative analysis of secretory lipase genomic arrangement was performed among *Moesziomyces* and related genera in MycoCosm (https://mycocosm.jgi.doe.gov/). Finally, comparative evolution (duplication and speciation events) of the *LipA* gene among members of *Ustilaginaceae* was carried out in EnsemblFungi (https://fungi.ensembl.org/index.html).

### Growth bioassays in oil-amended media

Growth bioassays on crude oil-amended media for culturable fungi obtained were conducted as previously described [81].

### Extracellular lipase assay

Extracellular lipase activity for the Trinidad strains was assessed using a modified Rhodamine agar plate method [101]. Briefly, NA consisting of olive oil (3% v/v), Rhodamine 6G solution (0.001% w/v), pH 7, supplemented with 50 mg/L each of streptomycin and tetracycline, were inoculated with the Trinidad strains, and incubated at 25 °C in the dark. As a control, un-inoculated plates were also prepared. Assays were performed in triplicate. This assay was also carried out on plates with crude oil (1–3% v/v) as a substitute for olive oil as the carbon source. The plates were examined under UV light and activity was determined by visual inspection for yellow-to-orange-coloured fluorescence [101–103].

## Supplementary Information

**Additional file 1.**

## Data Availability

All data generated or analysed during this study are included in this published article and its supplementary information files.

## References

[1] Abdel-Moghny T, Mohamed RSA, El-Sayed E, Aly SM, Snousy MG (2012). Removing of hydrocarbon contaminated soil via air flushing enhanced by surfactant. Appl Petrochem Res.

[2] Christofi N, Ivshina IB (2002). Microbial surfactants and their use in field studies of soil remediation. J Appl Microbiol.

[3] Fenibo EO, Ijoma GN, Selvarajan R, Chikere CB (2019). Microbial surfactants: the next generation multifunctional biomolecules for applications in the petroleum industry and its associated environmental remediation. Microorganisms..

[4] Primeia S, Inoue C, Chien M-F (2020). Potential of biosurfactants’ production on degrading heavy oil by bacterial consortia obtained from tsunami-induced oil-spilled beach areas in Miyagi, Japan. J Mar Sci Eng.

[5] Pereira JFB, Gudiña EJ, Costa R, Vitorino R, Teixeira JA, Coutinho JAP (2013). Optimization and characterization of biosurfactant production by *Bacillus subtilis* isolates towards microbial enhanced oil recovery applications. Fuel..

[6] Mohanty S, Jasmine J, Mukherji S. Practical considerations and challenges involved in surfactant enhanced bioremediation of oil. Biomed Res Int. 2013;2013. 10.1155/2013/328608. 10.1155/2013/328608 PMC3857904 24350261

[7] Cai Q, Zhang B, Chen B, Zhu Z, Lin W, Cao T (2014). Screening of biosurfactant producers from petroleum hydrocarbon contaminated sources in cold marine environments. Mar Pollut Bull.

[8] Santos DKF, Rufino RD, Luna JM, Santos VA, Sarubbo LA (2016). Biosurfactants: multifunctional biomolecules of the 21st century. Int J Mol Sci.

[9] Makkar RS, Cameotra SS, Banat IM (2011). Advances in utilization of renewable substrates for biosurfactant production. AMB Expr.

[10] Goto S, Sugiyama J, Iizuka H (1969). A taxonomic study of Antarctic yeasts. Mycologia..

[11] Spoeckner S, Wray V, Nimtz M, Lang S (1999). Glycolipids of the smut fungus *Ustilago maydis* from cultivation on renewable resources. Appl Microbiol Biotechnol.

[12] Ueda H, Mitsuhara I, Tabata J, Kugimiya S, Watanabe T, Suzuki K (2015). Extracellular esterases of phylloplane yeast *Pseudozyma Antarctica* induce defect on cuticle layer structure and water-holding ability of plant leaves. Appl Microbiol Biotechnol.

[13] Liu Y, Zou Z, Hu Z, Wang W, Xiong J. Morphology and molecular analysis of *Moesziomyces antarcticus* isolated from the blood samples of a chinese patient. Front Microbiol. 2019;10(254). 10.3389/fmicb.2019.00254. 10.3389/fmicb.2019.00254 PMC6384246 30828326

[14] Wang QM, Begerow D, Groenewald M, Liu XZ, Theelen B, Bai FY (2015). Multigene phylogeny and taxonomic revision of yeasts and related fungi in the *Ustilaginomycotina*. Stud Mycol.

[15] Hawksworth DL (2011). A new dawn for the naming of fungi: impacts of decisions made in Melbourne in July 2011 on the future publication and regulation of fungal names. IMA Fungus..

[16] Taylor JW (2011). One fungus = one name: DNA and fungal nomenclature twenty years after PCR. IMA Fungus..

[17] McNeill J, Barrie F, Buck W, Demoulin V, Greuter W, Hawksworth D (2012). International code of nomenclature for algae, Fungi and plants (Melbourne code).

[18] Kruse J, Doehlemann G, Kemen E, Thines M (2017). Asexual and sexual morphs of *Moesziomyces* revisited. IMA Fungus.

[19] Jezierska S, Claus S, Van Bogaert I (2018). Yeast glycolipid biosurfactants. FEBS Lett.

[20] Kitamoto D, Akiba S, Hioki C, Tabuchi T (1990). Extracellular accumulation of mannosylerythritol lipids by a strain of *Candida antarctica*. Agric Biol Chem.

[21] Morita T, Ishibashi Y, Hirose N, Wada K, Takahashi M, Fukuoka T (2011). Production and characterization of a glycolipid biosurfactant, mannosylerythritol lipid B, from sugarcane juice by *Ustilago scitaminea* NBRC 32730. Biosci Biotechnol Biochem.

[22] Boothroyd B, Thorn JA, Haskins RH (1956). Biochemistry of the *Ustilaginales*: Xii. Characterization of extracellular glycolipids produced by *Ustilago* sp. Can J Biochem Physiol.

[23] Deml G, Anke T, Oberwinkler F, Max Giannetti B, Steglich W (1980). *Schizonellin* A and B, new glycolipids from *Schizonella melanogramma*. Phytochemistry..

[24] Kakugawa K, Tamai M, Imamura K, Miyamoto K, Miyoshi S, Morinaga Y (2002). Isolation of yeast *Kurtzmanomyces* sp. I-11, novel producer of mannosylerythritol lipid. Biosci Biotechnol Biochem.

[25] Hewald S, Josephs K, Bölker M (2005). Genetic analysis of biosurfactant production in *Ustilago maydis*. Appl Environ Microbiol.

[26] Rau U, Nguyen LA, Schulz S, Wray V, Nimtz M, Roeper H (2005). Formation and analysis of mannosylerythritol lipids secreted by *Pseudozyma aphidis*. Appl Microbiol Biotechnol.

[27] Saika A, Koike H, Fukuoka T, Yamamoto S, Kishimoto T, Morita T (2016). A gene cluster for biosynthesis of mannosylerythritol lipids consisted of 4-O-β-D-mannopyranosyl-(2R,3S)-erythritol as the sugar moiety in a basidiomycetous yeast *Pseudozyma tsukubaensis*. PLoS One.

[28] Morita T, Konishi M, Fukuoka T, Imura T, Kitamoto HK, Kitamoto D (2007). Characterization of the genus *Pseudozyma* by the formation of glycolipid biosurfactants, mannosylerythritol lipids. FEMS Yeast Res.

[29] Hewald S, Linne U, Scherer M, Marahiel MA, Kämper J, Bölker M (2006). Identification of a gene cluster for biosynthesis of mannosylerythritol lipids in the basidiomycetous fungus *Ustilago maydis*. Appl Environ Microbiol.

[30] Arutchelvi JI, Bhaduri S, Uppara PV, Doble M (2008). Mannosylerythritol lipids: a review. J Ind Microbiol Biotechnol.

[31] Morita T, Fukuoka T, Imura T, Kitamoto D (2013). Accumulation of cellobiose lipids under nitrogen-limiting conditions by two ustilaginomycetous yeasts, *Pseudozyma aphidis* and *Pseudozyma hubeiensis*. FEMS Yeast Res.

[32] Morita T, Fukuoka T, Imura T, Kitamoto D (2013). Production of mannosylerythritol lipids and their application in cosmetics. Appl Microbiol Biotechnol.

[33] Kitamoto D, Morita T, Fukuoka T, Konishi M-a, Imura T (2009). Self-assembling properties of glycolipid biosurfactants and their potential applications. Curr Opin colloid. Interface Sci.

[34] Kitamoto H, Yoshida S, Koitabashi M, Yamamoto-Tamura K, Ueda H, Yarimizu T (2018). Enzymatic degradation of poly-butylene succinate-co-adipate film in rice husks by yeast *Pseudozyma Antarctica* in indoor conditions. J Biosci Bioeng.

[35] Ron EZ, Rosenberg E (2001). Natural roles of biosurfactants. Environ Microbiol.

[36] Wakamatsu Y, Zhao X, Jin C, Day N, Shibahara M, Nomura N (2001). Mannosylerythritol lipid induces characteristics of neuronal differentiation in PC12 cells through an ERK-related signal cascade. Eur J Biochem.

[37] Kurz M, Eder C, Isert D, Li Z, Paulus EF, Schiell M (2003). Ustilipids, acylated β-D-mannopyranosyl D-erythritols from *Ustilago maydis* and *Geotrichum candidum*. J Antibiot.

[38] Im JH, Nakane T, Yanagishita H, Ikegami T, Kitamoto D (2001). Mannosylerythritol lipid, a yeast extracellular glycolipid, shows high binding affinity towards human immunoglobulin G. BMC Biotechnol.

[39] Inoh Y, Kitamoto D, Hirashima N, Nakanishi M (2001). Biosurfactants of MEL-A increase gene transfection mediated by cationic liposomes. Biochem Biophys Res Commun.

[40] Kitamoto D, Isoda H, Nakahara T (2002). Functions and potential applications of glycolipid biosurfactants - from energy-saving materials to gene delivery carriers. J Biosci Bioeng.

[41] Kitamoto HK, Shinozaki Y, Cao X-h, Morita T, Konishi M, Tago K (2011). Phyllosphere yeasts rapidly break down biodegradable plastics. AMB Expr..

[42] Morita T, Koike H, Hagiwara H, Ito E, Machida M, Sato S (2014). Genome and transcriptome analysis of the basidiomycetous yeast *Pseudozyma Antarctica* producing extracellular glycolipids, mannosylerythritol lipids. PLoS One.

[43] Morita T, Ito E, Kitamoto HK, Takegawa K, Fukuoka T, Imura T (2010). Identification of the gene *PaEMT1* for biosynthesis of mannosylerythritol lipids in the basidiomycetous yeast *Pseudozyma Antarctica*. Yeast..

[44] Kitamoto D, Yanagishita H, Haraya K, Kitamoto HK (1998). Contribution of a chain-shortening pathway to the biosynthesis of the fatty acids of mannosylerythritol lipid (biosurfactant) in the yeast *Candida antarctica*: effect of β-oxidation inhibitors on biosurfactant synthesis. Biotechnol Lett.

[45] Henkel M, Hausmann R, Hayes DG, Solaiman DKY, Ashby RD (2019). Diversity and classification of microbial surfactants. Biobased surfactants synthesis, properties, and applications.

[46] Morita T, Koike H, Koyama Y, Hagiwara H, Ito E, Fukuoka T (2013). Genome sequence of the basidiomycetous yeast *Pseudozyma Antarctica* T-34, a producer of the glycolipid biosurfactants mannosylerythritol lipids. Genome Announc.

[47] Brandt SC, Ellinger B, van Nguyen T, Thi QD, van Nguyen G, Baschien C (2018). A unique fungal strain collection from Vietnam characterized for high performance degraders of bioecological important biopolymers and lipids. PLoS One.

[48] Jan A-H, Dubreucq E, Drone J, Subileau M (2017). A glimpse into the specialization history of the lipases/acyltransferases family of CpLIP2. Biochim Biophys Acta, Proteins Proteomics.

[49] Sandström AG, Wikmark Y, Engström K, Nyhlén J, Bäckvall J-E (2012). Combinatorial reshaping of the *Candida antarctica* lipase a substrate pocket for enantioselectivity using an extremely condensed library. Proc Natl Acad Sci U S A.

[50] Gotor-Fernández V, Busto E, Gotor V (2006). *Candida antarctica* lipase B: an ideal biocatalyst for the preparation of nitrogenated organic compounds. Adv Synth Catal.

[51] Saika A, Koike H, Yarimizu T, Watanabe T, Kitamoto H, Morita T (2019). Deficiency of biodegradable plastic-degrading enzyme production in a gene-deletion mutant of phyllosphere yeast, *Pseudozyma Antarctica* defective in mannosylerythritol lipid biosynthesis. AMB Expr..

[52] Kitamoto H (2019). The phylloplane yeast *Pseudozyma*: a rich potential for biotechnology. FEMS Yeast Res.

[53] Historical Facts on the Petroleum Industry of Trinidad and Tobago. 2021. https://www.energy.gov.tt/historical-facts-petroleum/. Accessed 29 July 2021.

[54] Le SQ, Gascuel O (2008). An improved general amino acid replacement matrix. Mol Biol Evol.

[55] Palidwor G, Reynaud EG, Andrade-Navarro MA (2006). Taxonomic colouring of phylogenetic trees of protein sequences. BMC Bioinform.

[56] Morita T, Konishi M, Fukuoka T, Imura T, Kitamoto D (2006). Discovery of *Pseudozyma rugulosa* NBRC 10877 as a novel producer of the glycolipid biosurfactants, mannosylerythritol lipids, based on rDNA sequence. Appl Microbiol Biotechnol.

[57] Morita T, Fukuoka T, Imura T, Kitamoto D (2009). Production of glycolipid biosurfactants by basidiomycetous yeasts. Biotechnol Appl Biochem.

[58] Keller NP, Turner G, Bennett JW (2005). Fungal secondary metabolism - from biochemistry to genomics. Nat Rev Microbiol.

[59] Habchi J, Tompa P, Longhi S, Uversky VN (2014). Introducing protein intrinsic disorder. Chem Rev.

[60] van der Lee R, Buljan M, Lang B, Weatheritt RJ, Daughdrill GW, Dunker AK (2014). Classification of intrinsically disordered regions and proteins. Chem Rev.

[61] Tompa P (2002). Intrinsically unstructured proteins. Trends Biochem Sci.

[62] Ma AD, Brass LF, Abrams CS (1997). Pleckstrin associates with plasma membranes and induces the formation of membrane projections: requirements for phosphorylation and the NH_2_-terminal PH domain. J Cell Biol.

[63] D’Auria JC (2006). Acyltransferases in plants: a good time to be BAHD. Curr Opin Plant Biol.

[64] Bartoszewska M, Opaliński Ł, Veenhuis M, van der Klei IJ (2011). The significance of peroxisomes in secondary metabolite biosynthesis in filamentous fungi. Biotechnol Lett.

[65] Freitag J, Ast J, Linne U, Stehlik T, Martorana D, Bölker M (2014). Peroxisomes contribute to biosynthesis of extracellular glycolipids in fungi. Mol Microbiol.

[66] Wada K, Koike H, Fujii T, Morita T (2020). Targeted transcriptomic study of the implication of central metabolic pathways in mannosylerythritol lipids biosynthesis in *Pseudozyma Antarctica* T-34. PLoS One.

[67] Płaza GA, Zjawiony I, Banat IM (2006). Use of different methods for detection of thermophilic biosurfactant-producing bacteria from hydrocarbon-contaminated and bioremediated soils. J Pet Sci Eng.

[68] Walter V, Syldatk C, Hausmann R, Sen R (2010). Screening concepts for the isolation of biosurfactant producing microorganisms. Biosurfactants.

[69] Siegmund I, Wagner F (1991). New method for detecting rhamnolipids excreted by *Pseudomonas* species during growth on mineral agar. Biotechnol Tech.

[70] Thavasi R, Sharma S, Jayalakshmi S. Evaluation of screening methods for the isolation of biosurfactant producing marine bacteria. J Pet Environ Biotechnol. 2011;1(2).

[71] Youssef NH, Duncan KE, Nagle DP, Savage KN, Knapp RM, McInerney MJ (2004). Comparison of methods to detect biosurfactant production by diverse microorganisms. J Microbiol Methods.

[72] Russell NJ, Harrisson P, Johnston IA, Jaenicke R, Zuber M, Franks F (1990). Cold adaptation of microorganisms. Philos Trans R Soc Lond Ser B Biol Sci.

[73] Margesin R (2000). Potential of cold-adapted microorganisms for bioremediation of oil-polluted Alpine soils. Int Biodeterior Biodegrad.

[74] Mikolasch A, Donath M, Reinhard A, Herzer C, Zayadan B, Urich T (2019). Diversity and degradative capabilities of bacteria and fungi isolated from oil-contaminated and hydrocarbon-polluted soils in Kazakhstan. Appl Microbiol Biotechnol.

[75] Martin-Sanchez PM, Becker R, Gorbushina AA, Toepel J (2018). An improved test for the evaluation of hydrocarbon degradation capacities of diesel-contaminating microorganisms. Int Biodeterior Biodegrad.

[76] Kitamoto D, Ikegami T, Suzuki GT, Sasaki A, Takeyama Y-i, Idemoto Y (2001). Microbial conversion of n-alkanes into glycolipid biosurfactants, mannosylerythritol lipids, by *Pseudozyma* (*Candida antarctica*). Biotechnol Lett.

[77] Becker F, Stehlik T, Linne U, Bölker M, Freitag J, Sandrock B (2021). Engineering *Ustilago maydis* for production of tailor-made mannosylerythritol lipids. Metab Eng Commun.

[78] da Silva AF, Banat IM, Giachini AJ, Robl D (2021). Fungal biosurfactants, from nature to biotechnological product: bioprospection, production and potential applications. Bioprocess Biosyst Eng.

[79] Higgins G, Saunders J (1974). Mud volcanoes-their nature and origin, contribution to the geology and paleobiology of the Carribean adjacent areas. Verhand Natur Geschechaft Bazel.

[80] Comeau PL. The vegetation surrounding mud volcanoes in Trinidad. Living World J Trinidad Tobago Field Nat Club. 1993;(Living World 1993-1994):17–27.

[81] Ramdass AC, Rampersad SN (2021). Diversity and oil degradation potential of culturable microbes isolated from chronically contaminated soils in Trinidad. Microorganisms..

[82] Juhasz AL, Britz M, Stanley G (1997). Degradation of fluoranthene, pyrene, benz [a] anthracene and dibenz [a, h] anthracene by *Burkholderia cepacia*. J Appl Microbiol.

[83] White TJ, Bruns T, Lee S, Taylor J, Innis MA, Gelfand DH, Sninsky JJ, White TJ (1990). Amplification and direct sequencing of fungal ribosomal RNA genes for phylogenetics. PCR protocols: a guide to methods and applications.

[84] Vilgalys R, Hester M (1990). Rapid genetic identification and mapping of enzymatically amplified ribosomal DNA from several *Cryptococcus* species. J Bacteriol.

[85] Rehner SA, Samuels GJ (1995). Molecular systematics of the Hypocreales: a teleomorph gene phylogeny and the status of their anamorphs. Can J Bot.

[86] Liu K-L, Porras-Alfaro A, Kuske CR, Eichorst SA, Xie G (2012). Accurate, rapid taxonomic classification of fungal large-subunit rRNA genes. Appl Environ Microbiol.

[87] Raja HA, Miller AN, Pearce CJ, Oberlies NH (2017). Fungal identification using molecular tools: a primer for the natural products research community. J Nat Prod.

[88] Katoh K, Standley DM (2013). MAFFT multiple sequence alignment software version 7: improvements in performance and usability. Mol Biol Evol.

[89] Hall TA (1999). Bioedit: a user-friendly biological sequence alignment editor and analysis program for windows 95/98/Nt.

[90] Tamura K, Stecher G, Peterson D, Filipski A, Kumar S (2013). MEGA6: molecular evolutionary genetics analysis version 6.0. Mol Biol Evol.

[91] Stothard P (2000). The sequence manipulation suite: JavaScript programs for analyzing and formatting protein and DNA sequences. BioTechniques..

[92] Almagro Armenteros JJ, Tsirigos KD, Sønderby CK, Petersen TN, Winther O, Brunak S (2019). SignalP 5.0 improves signal peptide predictions using deep neural networks. Nat Biotechnol.

[93] Lao DM, Arai M, Ikeda M, Shimizu T (2002). The presence of signal peptide significantly affects transmembrane topology prediction. Bioinformatics..

[94] Krogh A, Larsson B, von Heijne G, Sonnhammer ELL (2001). Predicting transmembrane protein topology with a hidden markov model: application to complete genomes. J Mol Biol.

[95] Möller S, Croning MDR, Apweiler R (2001). Evaluation of methods for the prediction of membrane spanning regions. Bioinformatics..

[96] Schneider TD, Stephens RM (1990). Sequence logos: a new way to display consensus sequences. Nucleic Acids Res.

[97] Crooks GE, Hon G, Chandonia J-M, Brenner SE (2004). WebLogo: a sequence logo generator. Genome Res.

[98] Gupta R, Jung E, Brunak S. Prediction of N-glycosylation sites in human proteins. DTU Bioinformat. 2004.

[99] Stamatakis A (2014). RAxML version 8: a tool for phylogenetic analysis and post-analysis of large phylogenies. Bioinformatics..

[100] Kozlov AM, Darriba D, Flouri T, Morel B, Stamatakis A (2019). RAxML-NG: a fast, scalable and user-friendly tool for maximum likelihood phylogenetic inference. Bioinformatics..

[101] Kouker G, Jaeger KE (1987). Specific and sensitive plate assay for bacterial lipases. Appl Environ Microbiol.

[102] Corbellini VA, Scroferneker ML, Carissimi M, Stopiglia CDO, Souza TF (2007). Comparision of lipolytic activity of *Sporothrix schenckii* strains utilizing olive oil-Rhodamine B and tween 80. Tecno-lógica Santa Cruz do Sul RS.

[103] Kuzkova N, Popenko O, Yakunov A (2014). Application of temperature-dependent fluorescent dyes to the measurement of millimeter wave absorption in water applied to biomedical experiments. Int J Biomed Imaging.

