# Supplementary material for: Detection and diversity of the mannosylerythritol lipid (MEL) gene cluster and lipase A and B genes of Moesziomyces antarcticus isolated from terrestrial sites chronically contaminated with crude oil in Trinidad
Source: BMC Microbiol. 2022 Feb 4;22:43. doi: 10.1186/s12866-021-02419-4 (PMC8815271; doi:10.1186/s12866-021-02419-4)
Supplement: Supplementary file 1 — Additional file 1. [file 12866_2021_2419_MOESM1_ESM.docx]

**Detection and diversity of mannosylerythritol lipid gene cluster (MEL) and Lipase A and B genes of *Moesziomyces antarcticus* isolated from natural oil seeps in Trinidad**

**Supplementary Tables**

Table S1. List of reference nucleotide sequences and GenBank accession data used in ITS phylogenetic analysis.

| **Accession No.** | **Species** | **Strain/Culture/Voucher** | **Origin** |
| --- | --- | --- | --- |
| AB089370 | *Pseudozyma rugulosus* | JCM10323 | Canada |
| MK027034 | *Moesziomyces* sp. | BRIP51843 | Australia |
| AF294697 | *Pseudozyma rugulosa* | CBS 170.88 | - |
| NR_155364 | *Moesziomyces rugulosus* | JCM 10323 | Canada |
| AB089362 | *Pseudozyma aphidis* | JCM10318 | Germany |
| NR_145336 | *Moesziomyces aphidis* | CBS 517.83 | Brazil |
| JN942666 | *Pseudozyma aphidis* | JCM 10318 | Germany |
| KY104282 | *Moesziomyces antarcticus* | CBS:5955 | Antarctica |
| NR_155406 | *Moesziomyces antarcticus* | CBS 5955 | Antarctica |
| JN942668 | *Pseudozyma antarctica* | JCM 10317 | Antarctica |
| AB089358 | *Pseudozyma antarctica* | JCM10317 | Antarctica |
| MN855218 | *Macalpinomyces* sp. | JMK-2019a (isolate) | Australia |
| NR_171250 | *Macalpinomyces collinsiae* | BRIP 67533 | Australia |
| KY104283 | *Moesziomyces parantarcticus* | CBS:10005 | Thailand |
| NR_130693 | *Moesziomyces parantarcticus* | JCM 11752 | Thailand |
| AB089356 | *Pseudozyma parantarctica* | M9956 | Thailand |
| NR_077129 | *Pseudozyma prolifica* | CBS 319.87 | - |
| AB089368 | *Pseudozyma prolifica* | JCM10319 | Canada |
| KY104687 | *Pseudozyma hubeiensis* | CBS:10077 | China |
| NR_137546 | *Pseudozyma hubeiensis* | AS 2.2493 | China |
| KY104690 | *Pseudozyma tsukubaensis* | CBS:6389 | Japan |
| NR_167941 | *Pseudozyma tsukubaensis* | JCM10324 | Thailand |
| NR_154065 | *Kalmanozyma brasiliensis* | CBS 13268 | Brazil |
| KF737866 | *Kalmanozyma brasiliensis* | GHG001 | Brazil |
| AB089366 | *Pseudozyma fusiformata* | JCM3931 | Thailand |
| KY104688 | *Pseudozyma pruni* | CBS:10937 | Taiwan |
| EU379942 | *Pseudozyma pruni* | BCRC34227 | Taiwan |
| NR_165983 | *Pseudozyma pruni* | CBS 10937 | Taiwan |
| NR_155686 | *Pseudozyma thailandica* | CBS 10006 | Thailand |
| KY104689 | *Pseudozyma thailandica* | CBS: 10006 | Thailand |
| AB089354 | *Pseudozyma thailandica* | M9957 | Thailand |
| KY105787 | *Ustilago abaconensis* | CBS:8380 | Bahamas |
| NR_132053 | *Ustilago nunavutica* | DAOM 91211 | - |
| NR_155995 | *Ustilago shanxiensis* | CBS 10075 | China |
| DQ008956 | *Pseudozyma shanxiensis* | AS 2.2523 (ex-type) | China |
| NR_154694 | *Anthracocystis grodzinskae* | KRAM F-57394 | Benin |
| KP297998 | *Anthracocystis grodzinskae* | KRAM:F-57394 | Benin |

Table S2. List of reference protein sequences and GenBank accession data used in Emt1p protein tree.

| **Accession No.** | **Product** | **Source** |
| --- | --- | --- |
| CCF52717 | uncharacterized protein UHOR_04876 | *Ustilago hordei* |
| SAM82152 | Erythritol-mannosyl-transferase involved in MEL production | *Ustilago bromivora* |
| SPO29778 | probable Erythritol-mannosyl-transferase involved in MEL production | *Ustilago trichophora* |
| SPO29099 | probable Erythritol-mannosyl-transferase involved in MEL production | *Ustilago trichophora* |
| CDI53946 | conserved hypothetical protein | *Melanopsichium pennsylvanicum* 4 |
| XP_011389468 | Erythritol-mannosyl-transferase involved in MEL production | *Ustilago maydis* 521 |
| XP_012190145 | glycosyltransferase | *Pseudozyma hubeiensis* SY62 |
| SJX63359 | Erythritol-mannosyl-transferase involved in MEL production | *Sporisorium reilianum* f. sp. *reilianum* |
| CBQ73522 | conserved hypothetical protein | *Sporisorium reilianum* SRZ2 |
| XP_029740445 | hypothetical protein EX895_002448 | *Sporisorium graminicola* (*Pseudozyma graminicola*) |
| CDR99457 | hypothetical protein | *Sporisorium scitamineum* |
| BAI77915 | mannosyltransferase, partial | *Moesziomyces antarcticus* (*Pseudozyma antarctica*) |
| GAC75887 | hypothetical protein PANT_19c00001 | *Moesziomyces antarcticus* T-34 |
| ETS61959 | mannosyltransferase | *Moesziomyces aphidis* DSM 70725 |
| XP_014653801 | glycosyltransferase | *Moesziomyces antarcticus* (*Pseudozyma antarctica*) |
| OJJ07838 (outgroup) | hypothetical protein ASPVEDRAFT_89071 | *Aspergillus versicolor* CBS 583.65 |

Table S3. List of reference protein sequences and GenBank accession data used in Mac1p protein tree.

| **Accession No.** | **Product** | **Source** |
| --- | --- | --- |
| ETS61961 | hypothetical protein PaG_03509 | *Moesziomyces aphidis* DSM 70725 |
| XP_014653798 | conserved hypothetical protein | *Moesziomyces antarcticus* (*Pseudozyma antarctica*) |
| GAC75889 | hypothetical protein PANT_19d00003 | *Moesziomyces antarcticus* T-34 |
| XP_012190147 | hypothetical protein PHSY_004141 | *Pseudozyma hubeiensis* SY62 |
| XP_011389467 | Acyltransferase involved in MEL production | *Ustilago maydis* 521 |
| CBQ73521 | conserved hypothetical protein | *Sporisorium reilianum* SRZ2 |
| SJX63358 | Acyltransferase involved in MEL production | *Sporisorium reilianum* f. sp. *reilianum* |
| CDU26160 | probable acyltransferase involved in MEL production | *Sporisorium scitamineum* |
| XP_029740446 | hypothetical protein EX895_002449 | *Sporisorium graminicola* (*Pseudozyma graminicola*) |
| SAM82155 | Acyltransferase involved in MEL production | *Ustilago bromivora* |
| CCF52716 | uncharacterized protein UHOR_04874 | *Ustilago hordei* |
| CDI53947 | conserved hypothetical protein | *Melanopsichium pennsylvanicum* 4 |
| SPO29100 | probable Acyltransferase involved in MEL production | *Ustilago trichophora* |
| SPO29779 | probable Acyltransferase involved in MEL production | *Ustilago trichophora* |
| OJJ07840 (outgroup) | hypothetical protein ASPVEDRAFT_143340 | *Aspergillus versicolor* CBS 583.65 |

Table S4. List of reference protein sequences and GenBank accession data used in Mac2p protein tree.

| **Accession No.** | **Product** | **Source** |
| --- | --- | --- |
| CDU23647 | related to L-gulonolactone oxidase | *Sporisorium scitamineum* |
| CDS02202 | hypothetical protein | *Sporisorium scitamineum* |
| CBQ67431 | related to L-gulonolactone oxidase | *Sporisorium reilianum* SRZ2 |
| SJX60067 | related to L-gulonolactone oxidase | *Sporisorium reilianum* f. sp. *reilianum* |
| XP_029742055 | hypothetical protein EX895_000068 | *Sporisorium graminicola* |
| XP_011386603 | hypothetical protein UMAG_10013 | *Ustilago maydis* 521 |
| XP_012190196 | hypothetical protein PHSY_004192 | *Pseudozyma hubeiensis* SY62 |
| CDI50991 | related to L-gulonolactone oxidase | *Melanopsichium pennsylvanicum* 4 |
| SPO19630 | related to L-gulonolactone oxidase | *Ustilago trichophora* |
| SPO20559 | related to L-gulonolactone oxidase | *Ustilago trichophora* |
| XP_041409244 | uncharacterized protein UHO2_00842 | *Ustilago hordei* |
| SAM72975 | related to L-gulonolactone oxidase | *Ustilago bromivora* |
| ETS63302 | hypothetical protein PaG_01578 | *Moesziomyces aphidis* DSM 70725 |
| GAC73276 | D-arabinono-1, 4-lactone oxidase | *Moesziomyces antarcticus* T-34 |
| XP_014659931 | conserved hypothetical protein | *Moesziomyces antarcticus* |
| XP_016294677 (outgroup) | D-arabinono-1, 4-lactone oxidase | *Kalmanozyma brasiliensis* GHG001 |

Table S5. List of reference protein sequences and GenBank accession data used in Mmf1p protein tree.

| **Accession No.** | **Product** | **Source** |
| --- | --- | --- |
| SJX62795 | related to mfs1-putative multidrug transporter | *Sporisorium reilianum* f. sp. *reilianum* |
| SJX63357 | related to Sge1-drug resistance protein | *Sporisorium reilianum* f. sp. *reilianum* |
| CBQ73520 | related to Sge1-drug resistance protein | *Sporisorium reilianum* SRZ2 |
| XP_029740447 | hypothetical protein EX895_002450 | *Sporisorium graminicola* (*Pseudozyma graminicola*) |
| CDR99456 | hypothetical protein | *Sporisorium scitamineum* |
| XP_011389466 | Major Facilitator involved in MEL transport | *Ustilago maydis* 521 |
| XP_012190148 | major facilitator super | *Pseudozyma hubeiensis* SY62 |
| CDI53948 | related to Sge1-drug resistance protein | *Melanopsichium pennsylvanicum* 4 |
| SAM82156 | related to Sge1-drug resistance protein | *Ustilago bromivora* |
| CCF52715 | related to Sge1-drug resistance protein | *Ustilago hordei* |
| ETS61962 | hypothetical protein PaG_03510 | *Moesziomyces aphidis* DSM 70725 |
| XP_014653797 | iron permease | *Moesziomyces antarcticus* (*Pseudozyma antarctica*) |
| GAC75890 | dehydrogenases with different specificities | *Moesziomyces antarcticus* T-34 |
| SPO29780 | related to Major Facilitator involved in MEL transport | *Ustilago trichophora* |
| SPO29102 | related to Major Facilitator involved in MEL transport | *Ustilago trichophora* |
| GAQ47252 (outgroup) | multidrug resistance protein Fnx1 | *Aspergillus niger* |
